# Challenges in the Development of Functional Assays of Membrane Proteins

**DOI:** 10.3390/ma5112205

**Published:** 2012-11-07

**Authors:** Louis Tiefenauer, Sophie Demarche

**Affiliations:** Paul Scherrer Institut, Villigen PSI CH-5232, Switzerland; E-Mail: sophie.demarche@psi.ch

**Keywords:** membrane protein, function, surface, lipid bilayers, silicon, polymer, microfluidics, review

## Abstract

Lipid bilayers are natural barriers of biological cells and cellular compartments. Membrane proteins integrated in biological membranes enable vital cell functions such as signal transduction and the transport of ions or small molecules. In order to determine the activity of a protein of interest at defined conditions, the membrane protein has to be integrated into artificial lipid bilayers immobilized on a surface. For the fabrication of such biosensors expertise is required in material science, surface and analytical chemistry, molecular biology and biotechnology. Specifically, techniques are needed for structuring surfaces in the micro- and nanometer scale, chemical modification and analysis, lipid bilayer formation, protein expression, purification and solubilization, and most importantly, protein integration into engineered lipid bilayers. Electrochemical and optical methods are suitable to detect membrane activity-related signals. The importance of structural knowledge to understand membrane protein function is obvious. Presently only a few structures of membrane proteins are solved at atomic resolution. Functional assays together with known structures of individual membrane proteins will contribute to a better understanding of vital biological processes occurring at biological membranes. Such assays will be utilized in the discovery of drugs, since membrane proteins are major drug targets.

## 1. Introduction

Life from a chemical point of view is the sum of concerted feedback-controlled chemical reactions taking place in separated compartments. Biological membranes consisting of lipid bilayers are the natural barriers of cells toward the outside and also of intracellular compartments such as mitochondria, the Golgi-complex and lysosomes. The bio-catalyzed reactions lead to synthesis and modification of molecules and are the base for cell division and consequently the growth of organisms. Proteins integrated within biological membranes execute therefore many vital functions. The transformation of solar radiation energy into energy-rich compounds such as ATP takes place at cell membranes. Communication between cells becomes possible by the binding of a hormone or a neurotransmitter molecule to a membrane receptor, resulting in an induced change of the receptor conformation, which consequently triggers a cascade of further reactions within the cell. Thus, membrane proteins are key players in signal transduction across cell membranes. They are attached to (monotopic) or integrated in lipid bilayers and their functionality is dependent on the presence of suitable lipids. A biochemical approach aims at investigating a chosen, purified membrane protein and understanding its function at a molecular level. About 7500 different genes are coding for membrane proteins, which is about 30% of the entire human genome [1]. However, resolution of the atomic structure of membrane proteins by X-ray crystallography is difficult, since the hydrophobic part of membrane proteins within the membrane hampers crystallization. About 350 unique structures of integral membrane proteins are known mid-2012 [2] and only about 10% of them are of mammalian origin. A known structure is the basis to model dynamic interactions with lipids, ligands or other proteins. For a full understanding functional assays or biosensors are required.

In this review technical and scientific aspects of reconstituted membrane proteins in artificial bilayers are presented. Starting with describing material structuring technologies, the impact of surfaces properties on lipid bilayer formation and stability is discussed. Integration of functional proteins in bilayers is addressed as a crucial and difficult step. The functional mechanism of different membrane proteins is outlined in details. The final aim of biochemical investigations is the understanding of the induced conformation changes of proteins at a molecular level. This knowledge is important for a rational drug design. Finally, it is demonstrated how such quantitative sensing devices contribute to better understand the structure-function relationship of preselected membrane proteins through multifactorial dynamic investigations. This overview should contribute to put supported lipid bilayers in a wider context.

## 2. Lipid Bilayers with Integrated Functional Membrane Proteins on Surfaces

Artificial bilayers should mimic biological membranes of a natural cell [3]. The cross-section profile of an integral membrane protein can be divided in three regions: the extracellular or outside part (*cis*), the hydrophobic transmembrane part and the intracellular or inside part (*trans*). Since the transmembrane part is hydrophobic, almost all membrane proteins need detergents or lipids to retain their 3D-structure in solution [4]. After expression in cell cultures or cell-free expression systems, the membrane protein of interest is solubilized by a suitable detergent, and subsequently purified. The purified protein can then be used directly in a binding assay or, after reconstitution into liposomes, for more sophisticated functional assays [5]. In such proteoliposomes many membrane proteins are reasonably stable and can be investigated [6,7,8,9]. The activity of a transporter for instance can be determined by measuring the uptake of a dyed cargo using a fluorimeter [10] or by quantifying transported dyes in microscopic investigations [11] into proteoliposomes. Electrochemical measurements such as patch-clamp techniques can only be successful, if a sufficiently high resistance is achieved between the two electrodes separated by the bilayer [12]. Electrochemical detection methods are relatively simple and sensitive, and electrodes can easily be integrated within sensing devices (Table 1). The development of practicable techniques to prepare *planar* lipid bilayers on surfaces, however, remains still a challenge even after more than twenty years of research [13]. Supported lipid bilayers have to be stable and the electrochemical detection simple [14,15,16]. When planar lipid bilayers are suspended in pores separating two compartments [17], many additional advantages are achieved: The functionality of the freely mobile membrane protein is retained, Giga-ohm-sealed bilayers allow sensitive detection of single molecules and the integration in microfluidic devices is relatively simple [18]. In this chapter concepts and materials used for planar lipid bilayer formation are presented.

**Table 1:** Materials used in biosensors of membrane proteins.

Material	Abbreviation	Material used for			Remarks
		Bilayer support	MFS	Detector	
anodic aluminum oxide	AAO	porous [19]	–	–	nanopores
gold	Au	tethered [15,20,21,22]	–	*P-L* ^1^ [23]	thiol-compounds
indium tin oxide	ITO	–	–	glass [24,25]	conducting plates
mercury	Hg	tethered [26]	–	drop [27]	–
platinum	Pt	–	–	*P-L* [28]; wire [29,30]	–
polycarbonate	PC	pores, *las* ^2^ [31,32] pores, *ion* ^3^ [33]	*mec* ^4^ [34]; *emb* ^5^ [35]	–	–
polylactic acid	PLLA	pores, *las* [32]	–	–	–
polyethyleneterephthalate	PETE	pores, *las* [32]	–	–	–
polymetylmetacrylate	PMMA	pore(s), *mec* [36,37] pore(s), *emb* [38]	*mec* [39,40] *emb* [38,41]	–	fitting layers [39]
polydimethylsiloxane	PDMS	pore, *emb* [42]	*cas* ^6^ [43]	–	–
polyvinylidenechloride	PVDC	pore, *mec* [34,35]	–	–	–
polytetrafluoroethylene	PTFE Teflon	pore *P-L* [30]	*mec* [31]	–	fitting layers [44]
		pore(s) *emb* [38], *las* [44]			
poly(p-xylylene)	parylene	pores, *P-L* [39,40]	–	–	nanopore [39]
photoresist, epoxy-based	SU-8	*P-L* [28,45]	–	–	–
quartz or glass	–	pore, *elc* ^7^ [46]; *ion* [47]	–	–	–
silicon	Si	pore(s), *P*-*L* *+* *dry* ^8^ [48] porous [49,50] pore(s), *ion* [51]	*wet* ^9^ *+* *dry* [48]	–	gold coated [49]
silicon nitride	Si_3_N_4_	pore(s), *e-b* ^10^ [52] *P-L* [53,54,55] pores, *C-L* ^11^*+ dry* [55,56]	–	–	polyimide [54] on glass [43]
silver/silver chlorid	Ag/AgCl	–	–	*cvd* ^12^ [35] wire [34,37,42,57]	

^1^ *P-L*: photolithography; ^2^ *las*: lasering; ^3^ *ion*: ion etching; ^4^ *mec*: mechanical treatment; ^5^ *emb*: embossing; ^6^ *cas*: cast; ^7^ *elc*: electrochemical treatment; ^8^ *dry*: dry etching; ^9^ *wet*: wet etching; ^10^ *e-b*: e-beam; ^11^ *C-L*: colloid lithography; ^12^ *cvd*: chemical vapor deposition.

Materials used to support tethered and free-standing planar lipid bilayers as well as for microfluidic systems (MFS) and detectors are listed. The different *fabrication techniques*, as used in the references given, are indicated as abbreviations in *italics* and specified in the footnote to the table. For further information see the text. Note that some *fabrication techniques* allow the generation of a single pore or arrays of pore(s), whereas some materials provide an undefined number of pores which is indicated as “porous” material.

### 2.1. Materials Used for Artificial Bilayers and Microfluidic Systems

A material suitable to bear lipid bilayers and transporting fluids meets the following requirements: (1) It is resistant to organic solvents; (2) Uptake of water is negligible; (3) Surface properties such as density, wettability and roughness are well defined or can be adjusted through chemical modifications of activated material; (4) Technologies are available for making structures of micro- and nanometer dimensions within the material and the resulting structures remain stable; (5) The properties of the material allow electrochemical detections; (6) For optical detection the material is transparent; (7) Assembly of the structured components to a microfluidic device is simple and can be achieved preferentially without glue or fittings; (8) The material is relatively cheap and it is available from reliable sources.

Obviously one specific material can hardly meet all these specifications perfectly. Simple and robust fabrication processes and reproducible preparation and detection methods are decisive factors to make bioanalytical devices for membrane proteins.

### 2.2. Silicon-Based Micro- and Nanofabrication Technologies

**Figure 1:**
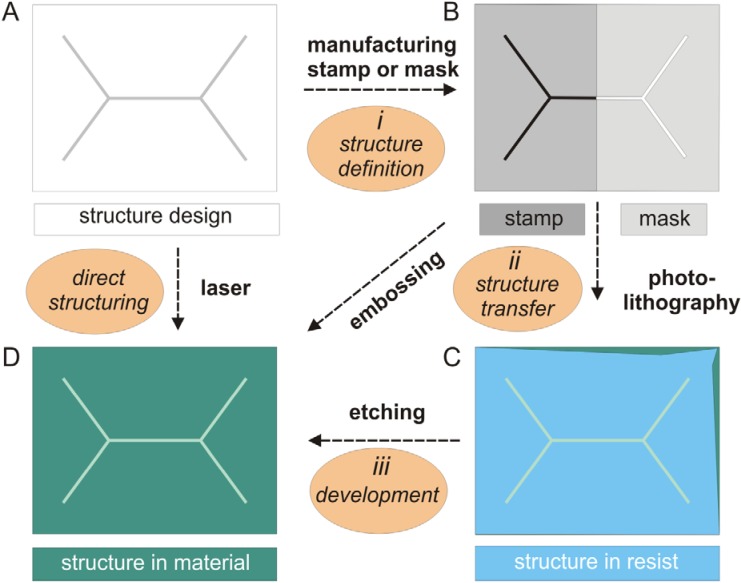
Structuring techniques for materials used in bioanalytics.

Various techniques for making structures such as pores or channels in micro- and nanodimensions have been developed [31,47,52,58,59,60]. In principle, micro- or nanostructuring of materials (Table 1) is realized in three steps: (1) Design of the structures; (2) transfer of this structure information to the target material and (3) development of the structure in the material (Figure 1). By using a laser, microstructures can directly be generated. The concept to assembly the micro- and nanostructured components to a functional device influences the performance, manufacturing and the cost of a bioanalytical device.

The design of a structure needed for microfluidics or as a support for free-standing bilayers (A) can be directly transferred by laser ablation into the material of interest, e.g., a polymer (D). For sequential high volume replicative fabrication processes a stamp is made, which consists of a solid material, e.g., brass and carries the negative raised structure of the design on a flat surface (B, left part). This negative structure is repetitively embossed into the target material, resulting in the wanted 3D-structures such as channels within the material (D). In highly parallel fabrication processes a metallic mask of negative (openings) structure of the design is first made (B, right part). This mask is used to transfer the targeted structure in a first step by shining light in photolithography to a resist (C). The illuminated areas of the resist are removed and 3D-structures such as channels or pores are etched into the material of choice (D). Using photolithographic techniques and silicon wafers a mass fabrication can be achieved.

Structuring of silicon wafers starts with a design of the wanted micro- or nanostructure and the subsequent production of the necessary masks. In clean rooms, the designed patterns are written in the resist on wafers using a mask and applying photolithographic processes followed by resist development and wet or dry etching processes (Figure 1). Structures as fluid channels or pores are made by these fabrication procedures which are highly reproducible and allow mass production [52]. For making nanostructures with dimensions beyond the limits of photolithography, focused ion beam or e-beam [61] facilities are needed. In the last two decades, arrays of nanopores in thin silicon nitride membranes have been made and used as supports for free-standing lipid bilayers [17,45,46,47,49,50,53,58,62,63,64,65]. The major advantage of nanometer-small pores is essentially a gain in stability of lipid bilayers suspended therein [55,64,66]. Free-standing lipid bilayers in pores with diameters of about one micrometer are sufficiently stable for many bioanalytical applications. For ion channels, which translocate ions across bilayers at a high rate, one single nanopore is sufficient, whereas for transporters of a three to four orders of magnitude lower translocation rate [5,67] arrays of pores will be required in order to achieve a detectable analyte concentration in the trans-compartment. Alternatively to silicon, aluminum oxide was utilized as a supporting material providing arrays of holes with a diameter of 50 nm [32,60]. Ion current flow mediated by proteins integrated in the stable and high resistant bilayers spanned over such nanopores can be detected electrochemically. A modification of the surface by hydrophobic silanes enhances the stability of suspended bilayers [19,64,68].

The ratio of pore length (L) to pore diameter (d) is defined as the aspect ratio. A low aspect ratio (L/d) of about 1 permits unhindered exchange of ions and molecules across supports, whereas the translocation of molecules is slowed down across bilayers suspended in long pores, *i.e.*, which have a high aspect ratio. Therefore, materials with nanopores of low aspect ratios are preferred for activity measurement of membrane transporters, where molecular diffusion is the limiting factor. In order to achieve a low aspect ratio of pores, the thickness of a material should be in the same range as the pore diameter. For pore diameters below 1 µm silicon nitride of 200 to 500 nm thickness is the material of choice. It is mechanically robust [54], and can be chemically derivatized like silica [64]. Pores in a wide range of diameters can be etched in such silicon nitride membranes and a single pore of a few nanometers in diameter has been made and used for DNA sequencing [69]. These pores in silicon nitride are open holes, in contrast to the structured surface of microbeads, which can be considered as nanowells of a few nanometers in diameter [56,59,70]. Functional bilayers covering these nanowells exhibit a high stability [70], but as it is true for tethered bilayer lipid membranes (tBLM), the transmembrane compartment is not accessible. Supports of aluminum [66], glass [71], quartz [46], and polymer foils have a minimum thickness of about 10 micrometers resulting in an aspect ratio of 10 or higher for nanopores, which is acceptable for ion channel measurements [47], but probably not for transporters.

### 2.3. Micro-Pore and Microfluidic Channels in Polymers

**Figure 2:**
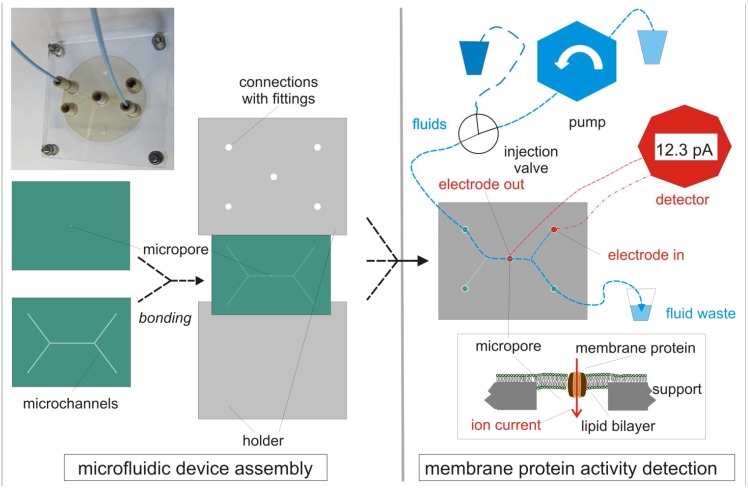
Microfluidics systems for membrane protein activity detection.

As mentioned above, a smart integration of the pore-containing component into a microfluidic system is required. In a first attempt pore (array) chips are usually connected to fluidic channels produced in slabs (see Table 1) made of polydimethylsiloxane (PDMS) [35,43,72,73]. Of course microchannels can also be etched directly into a silicon chip [45,48,74,75]. However, the key problem remains: To connect these micro-channels, which address the active sensing area on the chip, with the reservoirs containing the different fluids needed during analysis (Figure 2). Fluid transport and packaging are crucial issues concerning the fabrication of devices [74]. For many biosensor applications [76,77,78,79] patterns of the active area in the micrometer range [23,80,81] are required and this patterning process has to be made compatible with other fabrication processes.

**Figure 3:**
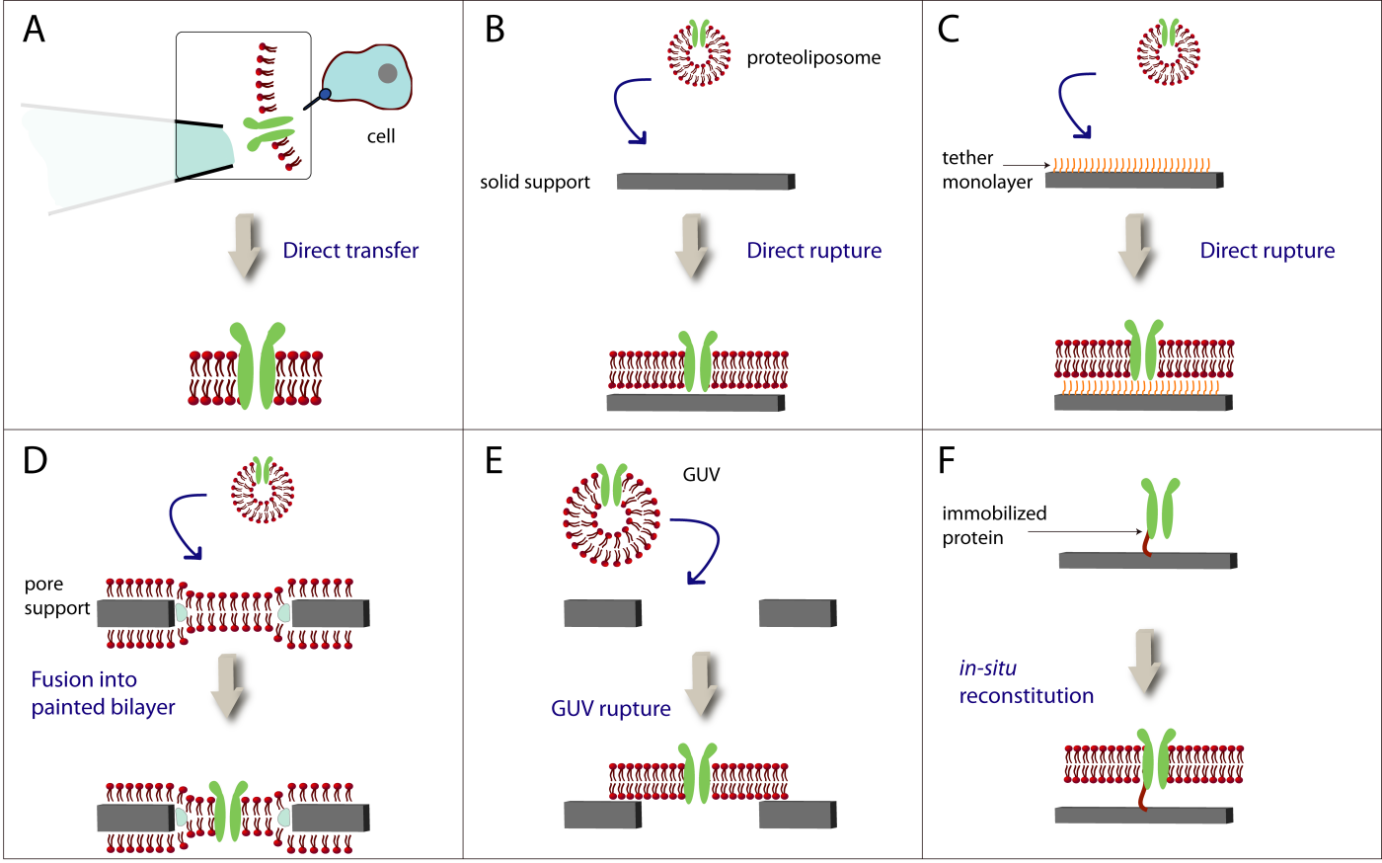
Protein integration methods.

As a typical example a PEEK polymer-based MFS is shown as recently reported [70]. In a thin polymer foil a micropore (10 µm diameter) is generated by laser ablation, in a thicker foil the microchannels are embossed at an elevated temperature. The two foils are tightly bonded to each other and two screwed PMMA-plates hold the resulting PEEK-MFS together. Fluids are transported through PEEK tubes, connected to NanoPorts^TM^, by a pump. One electrode is placed above the micropore, the other to an inlet. By adding a small volume (e.g., 10 µL) of a lipid dissolved in decane (dark blue) in a buffer carrier fluid (blue), a bilayer is formed in the micropore, resulting in a tremendous increase of resistance, as determined by an electrochemical detector. Various methods to insert the membrane protein of interest are shown in Figure 3. Protein activity is determined as a current in the pA-range, induced by an applied voltage (voltage-gated ion channel) or addition of a ligand (ligand-gated ion channels or activated transporter with an electrogenic substrate).

Various methods to integrate a membrane protein into a lipid bilayer have been reported. The protein can be directly transferred as a patch of a cell membrane using a glass pipette with a small orifice (A). For many methods proteoliposomes containing the protein of interest are used. Proteoliposomes can rupture directly on neat solid surfaces (B) or on chemically modified surfaces (C). They also fuse under specific conditions to a preformed lipid bilayer suspended in a pore (D). Giant unilamellar vesicles (GUVs), if positioned over a pore, rupture spontaneously resulting in pore-spanning lipid bilayers (E). For an oriented immobilization of the membrane protein to the surface, a His-tag (short red line) is utilized, and in a subsequent step, a lipid bilayer is formed (F).

Polymer materials are cheaper than silicon wafers and structuring methods for polymers are relatively simple compared to clean room technologies (Figure 1). Hot embossing [82] and injection molding techniques [83] are frequently used to produce polymer components also for biotechnological applications. Polymers are ideal for microfluidic devices in general and also for membrane protein assays, since this inexpensive material is biocompatible. Only few polymers meet all requirements listed above. Teflon [30,31,84] is inert, but too soft to make nanopores of low aspect ratios. In thin films of the frequently used poly(methyl methacrylate) (PMMA), micropores of 100 µm diameter [36] were generated and the foil was bonded at 110 °C to a PMMA plate which contain embossed micro-channels [38]. PDMS is widely used in research laboratories because it transforms structures with high fidelity and the casting procedures do not require an expensive clean room infrastructure. Sufficiently long stable bilayers were generated in a simple micro-chamber array addressed by micro-channels made in PDMS and the diffusion of the fluorescent dye calcein across a α-hemolysin pore was investigated [43]. However, PDMS has two severe disadvantages: (1) Due to its relatively low density it soaks solvents, which can contaminate subsequent fluids and (2) making nanopores of a low aspect ratio is not possible in this relatively soft material. However, the soaking property of PDMS has been utilized to induce lipid bilayers formation by painting a microchannel [42].

A suitable material for a microfluidic system, which is essentially assembled of micro-structured parts, should exhibit the following characteristics: It should be inert, dense, mechanically stable, showing low moisture uptake, and for electrochemical detection, exhibit a high electrical insulation and a low electrical capacitance. Polymers such as polyvinylidene chloride (PVDC), poly(p-xylylene) (parylene), polyether ether ketone (PEEK), polytetrafluoroethylen (PTFE) and polycarbonate [31] fulfill most of these requirements.

Ideally, the same material is utilized for making foils with micropore(s) and microfluidic channels [85]. This simplifies the sealing and the packaging (Figure 2). Several devices of various polymer materials have been reported. Micropores of 100 µm diameter have been generated in 50 µm thick PTFE foils using a laser beam and the micropore-containing foil has been clamped together with two PMMA-plates, containing the micro-channels, by the help of two sealing rubber gaskets resulting in a microfluidic system suitable for bilayer formation [44]. This microfluidic system consists of seven components and of different materials, what is too complicated for commercialization. In another microfluidic system a pore of about 20 µm diameter was burned in a 12.5 µm thick PVDC foil using a hot needle. The PVDC-foil was subsequently thermally bonded at 141 °C to a polycarbonate (PC) foil, in which 60 µm wide micro-channels have been embossed. The translocation of individual PEG-1500 molecules passing through α-hemolysin pores integrated in lipid bilayers suspended in the micropore could be demonstrated [35]. This polymer-hybrid microfluidic system demonstrates that rapid bilayer formation and subsequent multiple analysis of bilayers suspended in relatively large pores of 20 µm diameter is feasible. Another polymer-hybrid microfluidic system consists of a 5 µm thick parylene foil, in which a nanopore has been etched by applying processes adapted from silicon technology. The foil was integrated in a microfluidic system made of PMMA by thermal bonding at 120 °C [39]. In the pore of about 500 nm diameter a bilayer was painted and α-hemolysin has been integrated. In such small pores lipid bilayers with one integrated α-hemolysin pore were stable for 5 days. Very recently, a microfluidic device with a pore of about 10 µm diameter has been reported, built up of the high performance polymer PEEK [57]. The painted bilayers exhibited a high stability and allowed to monitor the opening and closing events of a single ion channel. This simple system consists only of two PEEK foils and can easily be adapted to high throughput analysis for membrane proteins, as reported for a parylene support [86]. In summary, polymeric materials are well suited to support free-standing lipid bilayers in micropores generated in thin foils and allow cost-effective and relatively simple fabrication and integration into microfluidic devices.

## 3. Lipid Bilayers with Functional Membrane Proteins

Biosensors are devices, in which a biological recognition element is in intimate contact to a detector. Differently to immune- or DNA-sensors [87], a functional membrane protein cannot be directly immobilized on the sensing surface without lipids or detergents. Membrane fragments or solubilized proteins can be immobilized and the binding of interacting proteins is measured optically [88]. For electrochemical functional assays as well as for AFM-imaging [89], however, the protein has to be integrated in a fluid lipid bilayer. In aqueous solutions lipids spontaneously self-assemble into liposomes consisting of defect-free bilayers. For quantitative measurements of protein-mediated ion flux across *planar* lipid bilayers, defect-free membranes over the entire sensing area are required as well. In addition, the protein-mediated activity has to be discriminated from the passive diffusion of ions. In this section different concepts are presented for preparing planar lipid bilayers and the influence of lipids on stability and functionality is discussed.

### 3.1. Formation of Lipid Bilayers on Solid Supports and Pores

Exactly 50 years ago, for the first time a technique was published, which described the method how artificial bilayers in small apertures can be made [90,91]. Later such painted free-standing black lipid bilayers were used to investigate valinomycin-mediated ion translocation between two compartments [92,93,94]. Since lipids are dissolved in organic solvents, residues thereof may be present in the pores after bilayer formation. Such residual organic solvent are not problematic for peptides, but such residues may denature more fragile membrane proteins. The limited stability of lipid bilayers suspended in such pores of about 100 µm diameter led to an intensive research on more stable alternatives. Bilayers on solid supports are obviously stable and factors determining the formation of lipid bilayers directly on solid surfaces have been intensively studied [16,95,96,97,98,99,100,101]. Proteoliposomes can be immobilized on solid surfaces [79,102], but determination of membrane protein related activities is then limited, as it is when cells are used. To monitor protein-mediated processes on *planar* lipid bilayers directly on detector surfaces is attractive and preparation methods for such solid supported bilayers were investigated for many years. Fusion of liposomes to hydrophobic surfaces is complex [97] and adsorption, rupture and spreading on hydrophilic surfaces can take hours [95]. Ca^2+^ promotes adsorption of negatively charged liposomes on hydrophilic silica [103] and saturated lipids are needed to achieve a sufficiently high stability in another case [96]. Buffer [104], lipid composition and surface charge [99,105] influence bilayer formation on silica supports. However, the major difficulty remains to keep membrane proteins functional in solid supported lipid bilayers. A suitable method may be to move lipid bilayers on solid supports [106,107] in microfluidic channels and thereby to induce a fusion with adsorbed vesicles from cells containing membrane proteins [108].

Fluidity of lipids is an indicator for bilayer formation and usually determined by the Fluorescence Recovery After Photobleaching (FRAP) method. For solid-supported bilayers the diffusion coefficient is in the range of 1–10 μm^2^ s^−1^ [108,109,110,111,112,113], as expected for planar lipid bilayers [114]. The mobility of the larger protein molecules in membranes is about one order of magnitude slower than that of the lipids [114]. A complete bilayer is a prerequisite for electrochemical detections and is checked by AFM-methods [98,115], quartz crystal microbalance [99] or confocal microscopy [101]. Successful bilayer formation depends on many factors such as temperature [116], vesicle size and composition, and surface density [99]. In conclusion, bilayer formation on solid supports has been widely investigated, but this approach is only of limited relevance to build up functional assays of membrane proteins. The transmembrane space of directly supported bilayers is about 1 nm wide [117], which is not enough to retain full mobility and function of many integral membrane proteins.

Therefore, since almost twenty years [118], tethered bilayer lipid membranes have intensively been investigated [3,16,119]. In tBLMs direct contact of the bilayer to the surface is avoided by the use of tethers, which provide enough space [120] for the intracellular part of integral membrane proteins [121]. The use of thiolpeptides [122,123,124] or thiolipids [21,125,126] on gold electrodes, ethylenglycol spacers [15,127,128,129] on silicon or a polyelectrolyte cushion [130] results in some cases in high insulating tBLMs [14,15,123,125,127], which are suitable to measure the activities of protein ion channels [126,129]. tBLMs were also achieved on aluminum oxide [112], on a lipid-polymer cushions [131,132] or on dextran immobilized on a surface using an antibody [133]. tBLMs are suitable to determine ion channel activities, however, the transmembrane space is also not directly accessible. Free access would be important to investigate transporters or protein-protein interactions, e.g., the typical G-protein coupled receptors (GPCR) interactions with intracellular G-protein [134].

In the last 10 years, free-standing bilayers suspended in very small pores were investigated. The stability of suspended bilayers in smaller pores is higher while both sides of the lipid bilayer remain accessible. Depending on the material used as discussed above, pore sizes can be achieved from about 2 nm [135] to macroscopic dimensions. The stability of suspended bilayers can be assessed by observing the spontaneous [64] or voltage-induced breakage. Already in the first publication on artificial bilayers [90], the electrical potential scanning was reported as a method to assess bilayer stability. Potential breakages above 300 mV usually indicate a sufficiently high stability [30,136]. Stability of bilayers in the range of 500 to 50 µm pore diameters remains unchanged and is rather low [30]. The breakdown voltage is about 250 mV for such bilayers. Below the pore diameter of about 50 µm, a steady increase of bilayer stability has been observed [30]. Beside the pore diameter, also the lipid composition and the density of integrated peptides or proteins influence the bilayer stability. Thus, a direct comparison of the stability of different preparations is difficult, since one single factor may be deciding. Pore sizes in the nanometer range in combination with different lipid compositions have been investigated systematically [64,137]. The little gain in bilayer stability below pore diameters of about 400 nm is probably not relevant, whereas the nature of the lipid used for bilayers plays a dominant role [64]. Since a more or less specific lipid composition is expected to be necessary to keep membrane proteins functional, a compromise will be necessary to achieve sufficiently high bilayer stability and retained functionality of the integrated membrane protein. In mammalian cells phospholipids are most abundant, whereas cholesterol makes lipid bilayers stiffer. Thus, phospholipids as, e.g., 1-palmitoyl, 2-oleoyl *sn*-glycero-3-phosphocholine (POPC) can be considered to be suitable lipids for biomimetic lipid bilayers. Phythanoyl lipids frequently used to generate stable bilayers [46,138,139] are probably not suitable to keep most ion channels and transporters functional as discussed below.

PEG-based hydrogel- [140] or polymer-cushion supported bilayers [141] in micropores can be considered as a compromise between free-standing and stable solid supported bilayers. Such bilayers are stable for days even in relatively large pores of 500 µm and the total resistivity is hardly enhanced by the presence of the supporting polymers [142]. At least such gel-like supports can be transparent for ions as confirmed by current measurements across α-hemolysin pores in bilayers supported by PEG-polymer [140].

### 3.2. Integration of Peptides in Lipid Bilayers

**Figure 4:**
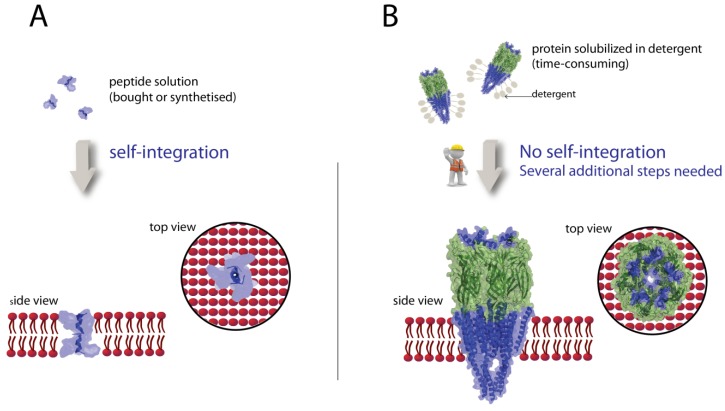
Peptidic and protein ion channels.

To characterize lipid bilayers formed on surfaces, solutions of self-integrating peptides are added and the resulting ions flux across the bilayer is monitored as a current. A prominent peptide is gramicidin [22], which forms a short alpha-helix, spanning one half of the bilayer sheet (Figure 4) [143,144]. When a peptide molecule in the outer leaflet of the bilayer is in line with one in the inner leaflet, a peptidic pore across the bilayer is intermediately formed, which can be detected as an ion current peak. A different mechanism has been found for the bee venom melittin. Monomers of the 26 amino acids peptide adsorb on the surface of a lipid bilayer and integrate therein. Subsequently these peptides form permanent pores [145], which can be measured as current peaks [21,62,141,146,147]. The translocation mechanism of ions across bilayers mediated by the cyclic peptide valinomycin is again different. This hydrophobic peptide is located within the bilayer and shuttles with relatively high selectivity potassium ions between two hydrophilic compartments. Despite its simple structure, it is very effective. The high unitary translocation rate of 10^4^, *i.e.*, one valinomycin molecule shuttles ten thousand K^+^ ions within one second [92]. Therefore, this peptide is frequently used to confirm bilayer functionality [64,148,149,150,151]. All the ion channel peptides have a relatively low molecular weight and consequently possess a limited defined tertiary structure, *i.e.*, can be easily handled in solutions without risking a loss of 3D-structure, *i.e.*, denaturation. In many reports they are called ion channels what is not wrong. However, there is a qualitative difference to integral *protein* ion channels, which have a molecular weight of some ten thousand daltons consisting of several domains and have a defined 3D-structure (Figure 4B). Such protein ion channels are much more fragile and difficult to handle. Therefore, it is important to recognize and point out that gramidicin, melittin, valinomycin, alamethicin are *peptidic* ion channels.

This figure illustrates that *peptidic* ion channels are far less complex than *protein* ion channels. A gramicidin A molecule (*pdb1MAG*) (A) is present as a single alpha helix structure in solution and two monomers inserts spontaneously into the lipid bilayers forming a pore which spans the bilayer. In contrast to peptides, membrane proteins as the pentameric acetylcholine receptor (*pdb2BG9*) (B) may be obtained through a series of preparation steps and detergents are needed for stabilization. The subsequent integration of reconstituted membrane proteins into a lipid bilayer is a difficult step.

### 3.3. Integration of Proteins in Lipid Bilayers

Due to the fragility of most membrane proteins, their integration into bilayers is considered as the critical step in biosensor development. Only very few integral membrane proteins self-integrate directly in lipid membranes [152,153]. Solutions of the bacterial α-hemolysin can be handled as a peptide solution and monomers integrate spontaneously into bilayers forming stable pores in a similar way as melittin. Therefore, α-hemolysin is frequently used to confirm functionality of artificial bilayers. The formation of the heptameric α-hemolysin pores takes a few seconds [154] up to some hours depending on the added concentration and the pore size [62]. The resulting proteolipid bilayers remain stable, but collapse, when the surface density of the pores in the membrane becomes too high [62], whereas the bilayer remains stable, if only one open pore is present [35]. Current across a pore can be detected electrochemically and simultaneously by total internal fluorescence reflection [11]. Molecules which are smaller than the 1.4 nm wide bottleneck of the hemolysin pore can freely pass through and their passages can be monitored as a drop in current at an applied voltage [155] This analytical system has been used to discriminate PEG-molecules by size [156]. Such a robust nanopore system has further been developed [157] into a commercial device for DNA-sequencing [158].

Further stable membrane proteins are protein pores [159], which derive from the outer membrane of bacteria, e.g., OmpF [65,153] or VhOmP [160]. As α-hemolysin pores these outer membrane pores are not selective and one open pore has a relatively high conductance of about 1 nS [160]. A mutated form of the membrane protein MspA with a bottleneck radius of 1.2 nm enables, similar as α-hemolysin pores, the sequencing of single-stranded DNA molecules at a rate of 28 ms per nucleotide with the help of a controller protein [161]. The realization of this innovative biosensor required long-term experience in structural biology for the design of a suitable recognition element, utilization of a classic enzyme from molecular biology and knowledge of preparation and detection of bilayers including the integrations of proteins, as discussed in this review. In another study OmpF pores present in giant unilamellar vesicles (GUV) were measured; sealing was achieved by applying a sub-pressure in a nano-pipette with the help of a manipulator [84]. Due to the small diameter of the orifice (243 nm), the achieved sealing resistance was very high allowing the measurement of the ion current across a single protein pore.

Much more demanding is the integration of functional ion channels and transporters into lipid bilayers (Figure 3). Unlike to self-integrating pore-forming membrane proteins, these integral membrane proteins need a permanent association with lipids or detergents to keep their structure [5]. The simplest way to achieve this is to transfer them directly from a cell by using a glass tip filled with agarose [162]. In this experiment, pore forming proteins and K^+^ channels have been recorded in a similar way as with patch-clamp techniques. However, many other proteins are present in natural cells, which may influence the targeted membrane protein.

To enable investigations of a pre-selected membrane protein of interest, several preparation steps are necessary, before functional analysis can be performed. In most cases the protein of interest is not present in a sufficiently high copy number in natural cells. If a plasmid coding for the protein is available, prokaryotic or eukaryotic cells are transformed or transfected, respectively, and may express the membrane protein in a sufficient quantity. The membrane fractions are solubilized, the protein is purified and reconstituted into a liposome or directly into a planar lipid bilayer [5]. Optimization of these steps is cumbersome and these tasks are often underestimated. Actually, the production of a sufficient quantity of solubilized or reconstituted membrane proteins is probably the rate limiting step in development of biosensors for most relevant membrane proteins. Consequently, only a few types of reconstituted ion channels have been investigated so far. Generally, the structure of prokaryotic membrane proteins is simpler than that one of the more relevant eukaryotic analogues as discussed below. Among other membrane proteins, ion channels from bacteria (NaChBac) [68,163] archaebacteria (KvAP) [9] and plants (KAT1) [164] have been reconstituted in proteoliposomes.

Fusion of individual proteoliposmes into bilayer membranes occurs sporadically. The probability of fusion can be enhanced by a suitable lipid composition [165,166], by using a combining hybridized DNA-sequence [167] or by destabilizing the proteoliposomes. A spontaneous destabilization is observed with GUVs [136] or when intermediate nystatin-peptide channels are formed building up an osmotic pressure upon addition of a high salt concentration [68,168,169]. The success rate of proteoliposome fusion has been enhanced and controlled by applying this technique and single ion channel events could be recorded [68]. A promising approach is to make use of the His-tag, which is present in most of recombinant membrane proteins. Detergent solubilized membrane proteins first bind to a NTA-derivatized matrix and the detergent molecules are then replaced by lipid molecules [170]. Such proteobilayers provide three advantages: The integrated membrane protein molecules are oriented, their surface density can be adjusted, and the use organic solvent is avoided. A high surface density of membrane proteins [78] as in natural cells [171] and their uniform orientation is essential [8,172] to build up transporter assays. Furthermore, as in the case of the spontaneous rupture of GUVs on surfaces [136], this solvent-free method prevents the denaturation of membrane proteins by residual organic solvent which may be present in the annulus, *i.e.*, the contacting area of painted bilayers to the rim of pores.

In summary, it is a big challenge to express and to integrate functional reconstituted eukaryotic membrane proteins such as ion channels and transporters in a sufficiently high density and this fact mainly limits the development of biosensors for relevant drug targets.

### 3.4. The Impact of Lipid Composition on Lipid Bilayer Stability and Protein Function

Several hundred different lipids occur in natural cells and this high variety may also have functional relevance. Lipids determine the mechanical properties of a cell membrane and the interaction of a specific lipid can affect the function of an integrated membrane protein. Lipids as ambiphilic molecules carry positive and negative charge units, which influence adsorption and fusion. Thus, a suitable lipid composition is important in order to achieve on one hand stable planar lipid bilayers as discussed above, and on the other hand, to retain the full functionality of proteins integrated therein.

Relatively little is known about the impact of lipids on the functionality of eukaryotic membrane proteins and which lipid compositions are suitable for biosensor applications [173]. The emerging field of lipidomics [174] analyzes the impact of lipids on cell functions in analogy to genomics, proteomics and glycomics. In our context both, the mechanical and functional aspects of lipids on membrane proteins [175] are crucial. Short chained phosphatidyl choline lipids as present in natural soy lecithin significantly reduce the stability, whereas cylindrical shaped lipids such as DOPE enhance it [64]. A high stability and sealing resistance as it is required for recording single ion channels is achieved, if two phythanoyl-chains are present in a lipid molecule as, e.g., in di-phythanoyl phosphatidyl choline (DPhPC) [138]. tBLM of this lipid [21] can host peptides [139] or α-hemolysin [63]. However, it is questionable if this plant lipid is suited to keep animal *eukaryotic* membrane proteins functional. The same reservation is made for stable bilayers achieved with polymerized lipids [139,176,177,178]. Phospholipids, e.g., POPC are the most abundant lipids of cells and many eukaryotic membrane proteins will probably remain active in artificial bilayers made of them. The steroid cholesterol providing stiffness to a natural membrane has also a functional role [179,180,181]. Preparations of biologically relevant rafts of lipids such as cholesterol [182] in planar lipid bilayers [49,183] will allow us to investigate the mechanism of cell adhesion [184] and function at predefined compositions [185]. Charged lipids such as di-oleoly phosphatidyl glycerol (DOPG), di-oleoyl phosphatidyl serine (DOPS) or di-oleoyl phosphatidyl ethanolamine (DOPE) are known to promote fusion of oppositely charged vesicles [186,187] and sphingolipids are involved in cell trafficking [182,188]. Charged lipids found in bacterial membranes [189] can also be used for artificial membranes to keep bacterial membrane proteins functional. Recently, the usefulness of planar lipid bilayers to investigate the interaction of a receptor with a drug has been demonstrated [185]. A tetracaine-induced conformational change of a reconstituted acetylcholine receptor (AChR) was determined by Fourier transform infrared (FTIR) difference spectroscopy in the presence of cholesterol [190]. It has to be kept in mind that the lipid composition in supported lipid bilayers formed by vesicle rupture may be different from that of the vesicles due to phase behavior of the mixed lipids [191].

## 4. Importance of Membrane Proteins in Biology and as Drug Targets

**Figure 5:**
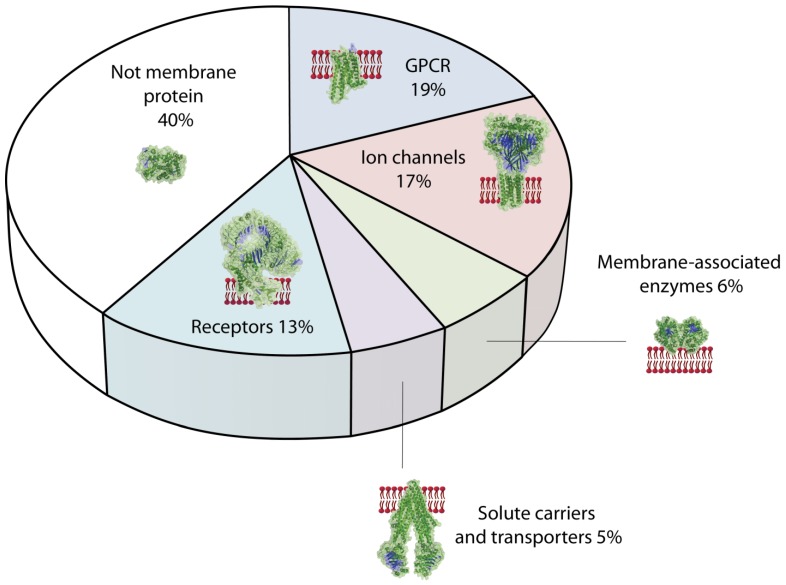
Human membrane proteins as drug targets.

In the last 50 years investigations of the structure and function of soluble proteins was promoted by technical achievements in molecular, cell and structural biology as well as by the tremendous progress in material sciences and instrumentation. Although the importance of membrane proteins was recognized early, the difficulty to retain their structure *in vitro* was the limiting factor to investigate them [192]. Biosensors and functional assays for many of the about 7500 different human membrane proteins would promote our understanding of biological processes. Since about 55% of all presently known over 430 drug targets are membrane proteins (Figure 5) [193] functional assays have also a high potential to promote the discovery of effective drugs. Here, a short overview of the different types of membrane proteins is given to illustrate the potential of functional assays for basic science and applications in bioanalytical tools.

Human membrane proteins of different classes represent about 60% of all protein drug targets: GPCRs are most prevalent, followed by ion channels and receptors. Membrane-associated enzymes, solute carriers and transporters are also important drug targets. The different classes are illustrated presenting a typical protein structure. GPCRs: adenosine receptor (*pdb3RFM*); ion channels: ASIC1a (*pdb2QTS*); receptors: toll-like receptor (*pdb3J0A*); solute carriers and transporters: p-glycoprotein (*pdb3g61*); membrane-associated enzymes: fatty acid amide hydrolase (*pdb1MT5*); not membrane protein: Human cytochrome P450 (*pdb1W0G*) [194].

### 4.1. Classification of Membrane Proteins Relevant as Drug Targets

From all human membrane proteins the integral or transmembrane proteins are the most interesting concerning function. This category of membrane proteins comprises cell adhesion proteins, receptors, ion channels, and transporters. For biosensor applications G-protein coupled receptors (GPCRs), ion channels and transporters are the most important membrane proteins (Figure 5).

All GPCRs consist of seven transmembrane helices and have been classified in 6 classes (A-F) according to structural and functional criteria. A specific GPCR can be activated by light, hormones or odorants resulting in the dissociation of the G (αβγ)-protein into the α- and the βγ-subunit. Both subunits are effectors for adenylcyclase or ion channels triggering the intracellular signaling cascade [134]. About half of the about 800 different GPCR-genes, which represent almost 4% of the total human genome, are coding for odorants and the other half for hormone receptors. More than 80% of GPCRs, including the odorant receptors, are grouped in class A, the rhodopsin-like receptors. Crystallization of fragile membrane proteins is difficult which is needed for structural resolution by X-ray crystallography. Recently, a structure of the human β_1_ adrenergic receptor could be solved at atomic resolution [195]. The importance of structural information [196] and functional assays [76,197] of these important classes cannot be overestimated, since GPCRs are major drug targets [194].

Ion channels, the second big category of drug targets, can further be classified according to their activation mechanism and selectivity. Roughly, ion channels are activated by a change in the membrane potential [198], by binding a ligand or by a mechanical stress. They are classified in voltage-gated [199], ligand-gated [200] or mechano-sensitive ion channels [201], respectively. Activation induces a conformational change of the membrane protein [202,203]. A selectivity for H^+^ (“proton”) [204], Na^+^ [205,206], K^+^ [207,208,209], Ca^2+^ [210], or Cl^−^ [211] is achieved through a selectivity filter [212], built up by the residues of a specific amino acid sequence at the entry of the transmembrane channel protein [213,214,215]. Ion channels control the ion flux across membranes [216] and can be considered as molecular switches in the propagation of the action potential of nerves. They are also important targets for drugs regulating heart and brain functions and for the treatment of pain [5]. Thus, the understanding of regulation and modulation of ion channel activities in details contributes to the development of potent drugs. Currently electrophysiological investigations of drug candidates are carried out on a patch of membranes from eukaryotic cells at a holding (clamped) potential. Such patch clamp techniques with cells bearing over-expressed ion channels are presently routinely carried out and have been automatized [217]. Planar patch clamp biosensors for specific eukaryotic ion channels of interest [218] will allow us to investigate the activation or inhibition mechanism, modulation by interacting proteins and contribute to identify drugs with less side effects through modulating membrane protein activities.

Transporters and carriers translocate charged or uncharged molecules across the cell membrane. Transporters regulate the uptake of nutrients as glucose in mammalian or ammonia [219] and water by aquaporins in plants [220]. Almost 400 different genes of ATP-binding cassette (ABC) transporters and solute carriers (SLC) have been identified in the human genome [221]. The gene products predominately are located in the intestine epithelia, blood-brain barrier, hepatocytes and kidney proximal tubules. Transporters such as P-glycoprotein (P-gp = MDR1) control the efflux of drugs and are responsible for multidrug resistance (MDR). Many drugs used in cancer therapy are substrates of this transporter. Reliable test systems specifically for P-gp [222] and many other ATP-transporters would enable investigations of the transport and to determine efflux rates of drug candidates. This knowledge will help to develop drugs, which are less prone to drug resistance [221].

Finally, a protein complex is mentioned here, which is not a drug target, but provides a mechanistic insight in the translocation of large molecules across membranes. The large nuclear pore complex [223] consisting of about 30 different proteins with a total molecular weight of 120 MDa, is present in the membrane of the nucleus. This protein pore regulates the transport of a cargo, especially RNA-polymers into the cytosol. The transport across the nuclear membrane is regulated by karyopherins (Kaps) [224]. The transport mechanism has been studied using a 30 nm pore in a solid silicon nitride coated with gold, to which phenylalanine- and glycine-rich nucleoporines are attached mimicking the situation of a natural pore [223]. This example shows how nanotechnological structures can probably contribute to the understanding of biological processes.

### 4.2. Prokaryotic *vs.* Eukaryotic Protein Structure

Prokaryotic 3D-structures of transporters are simpler than those of the eukaryotic analogues. However, due to the high structural similarity, mechanistic analysis, modeling and studies of the simpler membrane proteins can contribute to understand the relevant human membrane proteins. A few examples are given here to underline this statement.

The voltage-gated prokaryotic sodium channel (NaChBac) consists of 274 amino acids, six transmembrane helices (6TM), and forms a homotetramer in the membrane [225]. It plays a key function in pH homeostasis [226]. This relatively simple bacterial protein has structural similarities in the pore region to Ca^2+^ channels [227] and was also used as a simple model [163] for the more complex eukaryotic voltage-gated sodium channel Na(v)1.1, which consists of four different 6TMs domains. A highly resolved structure of this eukaryotic channel is still not yet available. Dysfunction of the human Na(v)1.1 channel are related to seizure and mutations therein can cause epilepsy [228]. Better knowledge of structures and functional mechanism of ion channels from bacteria to human can contribute to improved medical therapies.

A prominent example of a ligand-gated ion channel (LGIC) is the nicotinic acetylcholine receptor (nAChR) (see Figure 4B). This asymmetric receptor channel, consisting of four different subunits, forms a pentameric α_2_βγδstructure. It is located in the synapses of neurons and is activated by binding the neurotransmitter acetylcholine. The change of the membrane potential, the nerve signal, induces the release of the neurotransmitter from vesicles into the synaptic gap. The very short pulse time of 0.2 ms is achieved by the fast elimination of the neurotransmitter by acetylcholine esterase and the fast channel open-closing cycle [229]. Despite decades of research spent on this therapeutically important channel [230], many questions remain open. The structure of nAChR could not yet be solved at atomic-resolution [230]. Nevertheless, a detailed gating mechanism was proposed based on functional studies and confirmed by molecular modeling [231]. Recently, orthologs of this pentameric channel have been discovered in bacteria providing the first high resolution structure of an analogue ion channel [232]. The architectural structure, conformation analysis and functional studies from closely related receptor channels provide dynamic information about the activation mechanism. Drugs bind at allosteric site and lipids [190] modulate the activity of AChR. The combined efforts from structure resolution, functional studies on isolated membrane proteins [233], and dynamic molecular simulations pave the way to a full understanding and to a rational drug design.

### 4.3. Study of Protein—Protein Interactions at the Membrane

Reconstituted membrane proteins in free-standing lipid bilayers are accessible from both sides and can be studied at defined conditions. The binding of a ligand results in a conformational change of the membrane protein. In many cases two or more proteins are involved in signal transduction or electron transfer across membranes [234]. In order to study such complex protein-protein or protein-lipid interactions [235], artificial membrane devices will be helpful. Experiments can be carried out to investigate effects by addition of ligands, inhibitors or modulators as already demonstrated for the simpler pore forming proteins [34]. Furthermore, interactions occurring on the trans-side of the bilayer such as, e.g., binding of the natural protein inhibitor arrestin [236] to GPCRs can be investigated. The mechanical properties of such proteobilayer preparations [237,238] can also be characterized using AFM-techniques [239,240]. Free-standing membranes allow us to study the working mechanism of membrane protein complexes with effectors at a clamped voltage. Membrane protein activity modulating factors could be identified [241] using a defined molecular composition. In summary, functional assays of membrane proteins provide dynamic information needed for a better understanding of biological processes on membranes [242].

### 4.4. Biosensor for Drug Discovery

**Figure 6:**
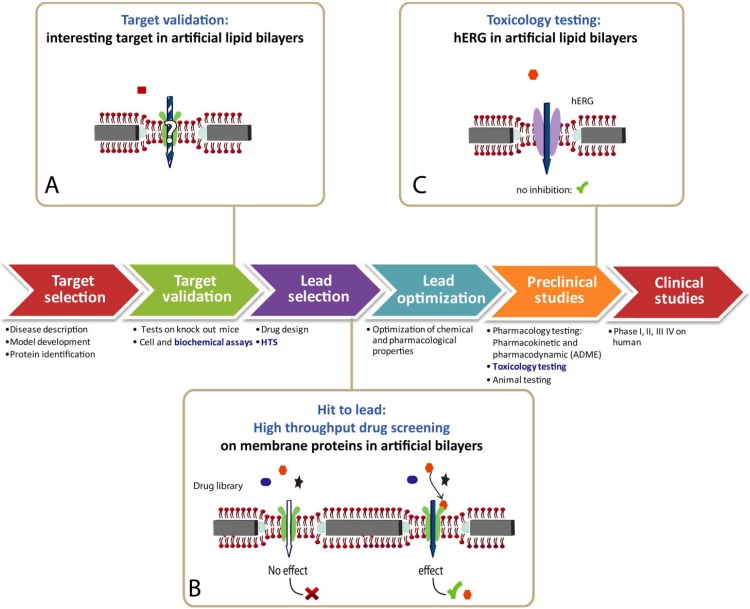
Drug discovery applications.

Biotechnology provides major tools to identify or validate drug targets (Figure 6) [243]. In order to reduce a waste of resources, hit identification and lead optimization at an early stage is crucial [193]. Beside the important contributions from structural and molecular biology, functional information from patch clamp on cells [244] promote the difficult endeavor to identify potent new drugs. In drug discovery, especially the detection of side effects is of pivotal importance. A potential drug compound blocking a specific K^+^ channel, coded by the human ether-à-go-go related gene (hERG) [245], provides a strong indication for other heavy and unacceptable side effects. Thus, hERG-tests are routinely carried out [246] using high throughput screening (HTS) technologies [217], which provides sufficient data in reasonable time [247]. Labor-intensive patch clamp techniques on cells have been automatized by several companies [5]. Artificial membranes [17], as discussed in this review, would provide some important advantages over cell-based assays and such HTS toxic assays using artificial membranes are highly desirable [248].

To our knowledge, presently no HTS of reconstituted membrane proteins is on the market. Knowledge transfer from multidisciplinary biosensor research of academia to small biotechnology firms is difficult [249]. Due to this fact only a few biosensors have been successfully commercialized. Biosensors are predominantly sold for monitoring glucose concentrations in the treatment of diabetes. Compared to the rather simple electrochemical enzymatic glucose oxidase measurement, assays for membrane protein integrated in lipid membranes are far more complex and their development is still in an early stage [18]. A close collaboration of academic together with industrial partners is the best way to promote curiosity-driven knowledge into a market-driven biosensor product [249].

Figure 6: Simplified subsequent phases of the drug discovery process are represented as a series of arrows. Biochemical assays provide functional information of the drug target (A). Among a plethora of precursor drug candidates, which bind to a drug target such as a membrane protein, a lead compound is selected using high throughput screening (HTS) methods (B). The pharmacology of a selected lead is further optimized in the ADME (adsorption, distribution, metabolism, excretion) procedure (C). Toxicological effects potentially occurring *in vivo* are assessed by monitoring the inhibition of the potassium ion channel hERG at an early stage. Currently, cell-based functional assays are prevalent. They could be replaced in the future by HTS functional assays using membrane proteins integrated in engineered lipid bilayers.

## 5. Information Expected from Functional Devices

Knowledge of the atomic structure of membrane proteins is the base to understand biological processes. Thus, structural biology of membrane proteins were promoted in the past and the solved structures from a few GPCRs [250] ion channels [230] and transporters [251] opened an absolutely fantastic detailed view on the processes, how signals are transduced across biological membranes. The electrophysiological observation of activation, inhibition or modulation can be assigned to specific large or small conformation changes of the membrane protein. Simulation of lipid-protein interactions [235] can confirm mechanistic explanations of functions and reveal driving forces for the observed structural changes. Since lipids influence the properties of the membrane or can specifically interact with a specific integral membrane protein, experimental setups of bilayers with defined lipid compositions are useful. Such devices allow us to investigate effects resulting upon activation, stimulation or inhibition by measuring quantitatively ion channel signals. Determination of the impact of site-specific mutagenesis in functional membranes proteins contribute to identify structural entities, which are related to function. To illustrate the potential of such functional devices some structure-function relationship studies are presented.

### 5.1. Working Mechanism of GPCRs

**Figure 7:**
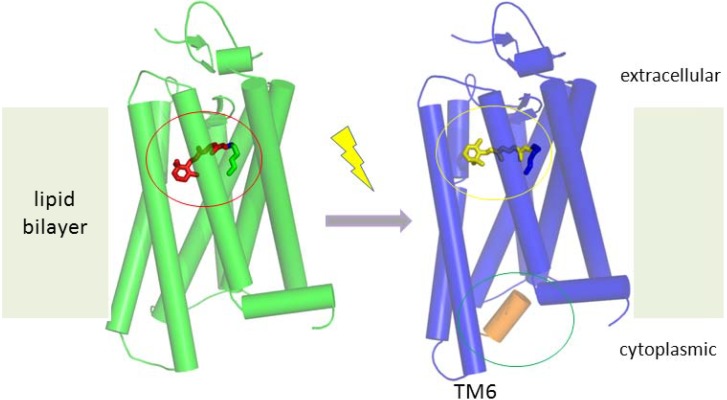
Structure-function relationship of membrane proteins.

Bacteriorhodopsin (BRh) is one of the first investigated membrane proteins, since it was available in sufficiently high quantities from large natural bacterial cultures. Crystallization of seven transmembrane helices (7 TM) proteins, despite their relatively low molecular weight, is difficult due to the mentioned hydrophobic transmembrane region. By using the mono-olein lipid, which spontaneously forms a lipid cubic phase, BRh integrated therein accumulate to 2D-crystals resulting in high structure resolution of BRh using X-ray crystallography [252]. The mechanism of proton translocation within the integral membrane protein across a membrane has been investigated and BRh was already twelve years ago the best understood membrane protein [253]. Soon later, the structure of mammalian rhodopsin [254] has been published and recently the activation mechanism for this membrane protein could be elucidated: The light-induced translocation of the β-ionone ring of the retinal molecule results in a rotation of transmembrane helix 6 (TM6) of the constitutively active mutant rhodopsin (Figure 7) [255].

GPCRs are important drug targets and the activation of rhodopsin as a typical representative is shown. Using X-ray crystallography light-induced conformation changes of stabilized eukaryotic mutants have been resolved at atomic resolution. Due to the light-induced conformation change from 11-*cis* (red circle) to all-*trans* (yellow circle) of the covalently bound retinal, the rigid transmembrane helix 6 (TM6) of rhodopsin is slightly rotated, resulting in opening of a cavity on the transmembrane side which enables the binding of the G-protein (green circle, an alpha-helical peptide part of the G-protein is shown as an orange cylinder), resulting in phosphorylation and intracellular cascade reactions [255].

Note that this mechanistic information was drawn from structures of proteins crystallized in discrete functional states. A prerequisite for this achievement was the development of new strategies to improve the success rate of structure resolution of the delicate GPCRs [256]. In order to better understand specificity and signal transduction of ligand-binding GPCRs, further structural information from proteins in different states of activation is needed. The over 300 different GPCRs bind a large repertoire of different natural molecules and odorants, however, their conformational change upon activation is highly conserved [256]. Again, structural information of a GPCR-drug target is indispensable to understand function [196]. Functional assay of reconstituted GPCRs [257], will allow us to investigate further the dynamic regulation by natural inhibitors or the modulation by pharmaceutical compounds. Solubilization [257] and integration of the GPCRs in nanodiscs [258] or in planar lipid bilayers provide the base for *in vitro* tests needed to investigate protein functionality [259] and monitor ligand-protein interactions.

### 5.2. Detection Methods for Membrane Protein Activities

Electrophysiological techniques are simple and best suited to quantify ion channel activities. The translocation activity of P-gp or other proteins can also been detected electrochemically, if the transported molecules are charged. However, due to the lower unitary transport rates of transporters, the passive diffusion of the species of interest across membranes cannot be neglected and a tight bilayer is essential. Using surface plasmon resonance (SPR) techniques, optical-active compounds accumulated in the trans-compartment can be quantified. Optical methods are also useful to detect binding of optically detectable ligands on reconstituted GPCRs [133] immobilized on surfaces [260]. Beside the frequently used optical and electrochemical detection, sensitive infrared spectroscopy [93,261] and enhanced Raman scattering [262] have been reported to monitor interactions of membrane proteins. For these detection methods, sealing resistance of the bilayer is not the critical factor. Optical and fluorescence techniques will offer excellent experimental flexibility to monitor complex processes and dynamic interactions occurring at cell membranes in a defined setup.

### 5.3. Contribution of Artificial Systems to the Understanding of Cell Functions

Debates on the significance of *in vitro* systems for cell processes will probably never end. The question is not, if valuable knowledge can be best acquired through investigation of natural cells or from reconstituted membrane proteins. Rather, the limitations of each approach better their contribution to understand biology should be recognized. A picture or a blueprint of a machine is in most cases not sufficient to understand what the machine is made for. Whole cells are of course more complex than man-made machines and elucidation of their functions do require many different complementary techniques. Quantitative ion flux measurements in the presence of ligands are performed to identify and to rank potent binders by using patch clamp techniques for cells with overexpressed channels. To which extent such cells reflect the behavior of natural cells, remains open. Furthermore, the complex interaction with unknown cell components is out of control. By microscopic methods a protein or organelle of interest can be precisely localized at a molecular level [263] within a cell and the induced changes can be monitored over time using fluorescent markers. The modification of the protein by a green fluorescence protein (GFP)-domain or a smaller fluorescent dye is assumed to have minimal influence on molecular interactions and the related functions. In summary, cell assays are important to visualize functions, but are not sufficient to fully understand mechanisms and processes.

For a detailed understanding of biological processes, further bottom-up approaches are necessary. Structures at atomic resolution provide the architecture of a molecular machine in a discrete state and molecular dynamic simulations can corroborate assumed functional steps. Since for protein crystallization truncated or stabilized protein fragments are frequently used, the conformational changes occurring in a full-length protein and the importance of flexible loops are out of the focus and remain obscure. Despite of these limitations, structural biology provides the essential base for understanding molecular processes of biology.

Finally, biochemistry provides complementary information. In biochemistry first a functional unit is isolated or produced (expressed) using molecular biological methods, followed by investigations under controlled conditions using dedicated instruments. Quantitative measurements of activation-related changes of the conformation or electronic status of a membrane protein with high temporal resolution provide essential information about dynamic processes. In biosensors such an experimental setup is further developed aimed at achieving a reliable, sensitive and robust analytical system for a specific application. A biosensor essentially combines a functional biological unit to a detector and can be considered as a highly sophisticated bio-analytical device.

## 6. Outlook

Progress in biosensor was not as fast as anticipated many years ago. Especially biosensors for membrane protein are difficult to realize and only a few prototypes have been developed. Not just “technical” problems are limiting. The identification of relevant factors for a sufficiently high bilayer stability *and* membrane protein functionality was in the focus in the past. However, a smart concept is needed, which includes all aspects of material properties, fabrication processes and assembly of components. Fabrication of sensors and devices should be simple and inexpensive and preparation and detection methods should be reliable and automatized. The aim of this review is to make aware that solving problems of bilayer preparation and signal detection is not sufficient. The membrane protein of interest is the key component, which is fragile and needs a suitable environment. This sensing surface has to be addressed by a fluidic system, which enables the addition of effectors and a rapid change of experimental conditions. A sensitive detection with low noise to monitor activation-related signals remains a challenge. In the past, emphasis was put on materials, surface chemistry and detection method using model membrane proteins. However, for a specific application dedicated engineered membrane proteins are required. Biosensors for hundreds of relevant membrane proteins can be made and the focus in the future will be put on all aspects concerning the biological recognition element [264].

## References

[1] Fagerberg L., Jonasson K., von Heijne G., Uhlen M., Berglund L. (2010). Prediction of the human membrane proteome. Proteomics.

[2] White S. Membrane proteins of known structures. http://blanco.biomol.uci.edu/mpstruc/listAll/list.

[3] Nielsen C.H. (2009). Biomimetic membranes for sensor and separation applications. Anal. Bioanal. Chem..

[4] Seddon A.M., Curnow P., Booth P.J. (2004). Membrane proteins, lipids, and detergents: Not just a soap opera. Biochem. Biophys. Acta.

[5] Demarche S., Sugihara K., Zambelli T., Tiefenauer L., Vörös J. (2011). Techniques for recording reconstituted ion channels. Analyst.

[6] Williams T.L., Vareiro M.M.L., Jenkins A.T. (2006). Fluorophore-encapsulated solid-supported bilayer vesicles: A method for studying membrane permeation processes. Langmuir.

[7] Pick H., Schmid E.L., Tairi A.P., Ilegems E., Hovius R., Vogel H. (2004). Investigating cellular signaling reactions in single attoliter vesicles. J. Am. Chem. Soc..

[8] Yanagisawa M., Iwamoto M., Kato A., Yoshikawa K., Oiki S. (2011). Oriented reconstitution of a membrane protein in a giant unilamellar vesicle: Experimental verification with the potassium channel KcsA. J. Am. Chem. Soc..

[9] Aimon S., Manzi J., Schmidt D., Larrosa J.A.P., Bassereau P., Toombes G.E.S. (2011). Functional reconstitution of a voltage-gated potassium channel in giant unilamellar vesicles. PLoS One.

[10] Lu P.H., Liu R.H., Sharom F.J. (2001). Drug transport by reconstituted P-glycoprotein in proteoliposomes—Effect of substrates and modulators, and dependence on bilayer phase state. Eur. J. Biochem..

[11] Heron A.J., Thompson J.R., Cronin B., Bayley H., Wallace M.I. (2009). Simultaneous measurement of ionic current and fluorescence from single protein pores. J. Am. Chem. Soc..

[12] Hamill O.P., Marty A., Neher E., Sakmann B., Sigworth F.J. (1981). Improved patch-clamp techniques for high resolution current recording from cells and cell-free membrane patches. Pflügers Arch..

[13] Sackmann E. (1996). Supported membranes: Scientific and practial applications. Science.

[14] Vockenroth I.K., Fine D., Dodobalapur A., Toby A., Jenkins A., Köper I. (2008). Tethered bilayer lipid membranes with giga-ohm resistance. Electrochem. Commun..

[15] Köper I. (2007). Insulating tethered bilayer lipid membranes to study membrane proteins. Mol. Biosyst..

[16] Anrather D., Smetazko M., Saba M., Alguel Y., Schalkhammer T. (2004). Supported membrane nanodevices. J. Nanosci. Nanotechnol..

[17] Janshoff A., Steinem C. (2006). Transport across artificial membranes—An analytical perspective. Anal. Bioanal. Chem..

[18] Suzuki H., Takeuchi S. (2008). Microtechnologies for membrane protein studies. Anal. Bioanal. Chem..

[19] Lazzara T.D., Kliesch T.T., Janshoff A., Steinem C. (2011). Orthogonal functionalization of nanoporous substrates: Control of 3D surface functionality. ACS Appl. Mater. Interfaces.

[20] Rossi C., Chopineau J. (2007). Biomimetic tethered lipid membranes designed for membrane-protein interaction studies. Eur. Biophys. J. Biophys. Lett..

[21] He L.H., Robertson J.W.F., Li J., Karcher I., Schiller S.M., Knoll W., Naumann R. (2005). Tethered bilayer lipid membranes based on monolayers of thiolipids mixed with a complementary dilution molecule. 1. Incorporation of channel peptides. Langmuir.

[22] Cornell B.A., Braach-Maksvytis L.B., King L.G., Osman P.D.J., Raguse B., Wieczorek L., Pace R.J. (1997). A biosensor that uses ion-channel switches. Nature.

[23] Han X., Critchley K., Zhang L., Pradeep S.N.D., Bushby R.J., Evans S.D. (2007). A novel method to fabricate patterned bilayer lipid membranes. Langmuir.

[24] Estes D.J., Lopez S.R., Fuller A.O., Mayer M. (2006). Triggering and visualizing the aggregation and fusion of lipid membranes in microfluidic chambers. Biophys. J..

[25] Chu L.K., Yen C.W., El-Sayed M.A. (2010). Bacteriorhodopsin-based photo-electrochemical cell. Biosens. Bioelectron..

[26] Becucci L., Moncelli M.R., Guidelli R. (2006). Impedance spectroscopy of OmpF porin reconstituted into a mercury-supported lipid bilayer. Langmuir.

[27] Becucci L., Guidelli R. (2007). Kinetics of channel formation in bilayer lipid membranes (BLMs) and tethered BLMs: Monazomycin and melittin. Langmuir.

[28] Aghdaei S., Sandison M.E., Zagnoni M., Green N.G., Morgan H. (2008). Formation of artificial lipid bilayers using droplet dielectrophoresis. Lab Chip.

[29] Quist A.P., Chand A., Ramachandran S., Daraio C., Jin S., Lal R. (2007). Atomic force microscopy imaging and electrical recording of lipid bilayers supported over microfabricated silicon chip nanopores: Lab-on-a-chip system for lipid membranes and ion channels. Langmuir.

[30] Mayer M., Kriebel J.K., Tosteson M.T., Whitesides G.M. (2003). Microfabricated teflon membranes for low-noise recordings of ion channels in planar lipid bilayers. Biophys. J..

[31] O'Shaughnessy T.J., Hu J.E., Kulp J.L., Daly S.M., Ligler F.S. (2007). Laser ablation of micropores for formation of artificial planar lipid bilayers. Biomed. Microdevices.

[32] Dhoke M.A., Ladha P.J., Boerio F.J., Lessard L.B., Malinowska D.H., Cuppoletti J., Wieczorek D.S. (2005). Porous membranes for reconstitution of ion channels. Biochim. Biophys. Acta.

[33] Favero G., D'Annibale A., Campanella L., Santucci R., Ferri T. (2002). Membrane supported bilayer lipid membranes array: Preparation, stability and ion-channel insertion. Anal. Chim. Acta.

[34] Shao C.R., Sun B., Colombini M., DeVoe D.L. (2011). Rapid microfluidic perfusion enabling kinetic studies of lipid ion channels in a bilayer lipid membrane chip. Ann. Biomed. Eng..

[35] Hromada L.P., Nablo B.J., Kasianowicz J.J., Gaitan M.A., DeVoe D.L. (2008). Single molecule measurements within individual membrane-bound ion channels using a polymer-based bilayer lipid membrane chip. Lab Chip.

[36] Suzuki H., Tabata K.V., Noji H., Takeuchi S. (2006). Highly reproducible method of planar lipid bilayer reconstitution in polymethyl methacrylate microfluidic chip. Langmuir.

[37] Funakoshi K., Suzuki H., Takeuchi S. (2006). Lipid bilayer formation by contacting monolayers in a microfluidic device for membrane protein analysis. Anal. Chem..

[38] Sandison M.E., Morgan H. (2005). Rapid fabrication of polymer microfluidic systems for the production of artificial lipid bilayers. J. Micromech. Microeng..

[39] Kawano R., Osaki T., Sasaki H., Takeuchi S. (2010). A polymer-based nanopore-integrated microfluidic device for generating stable bilayer lipid membranes. Small.

[40] Le Pioufle B., Suzuki H., Tabata K.V., Noji H., Takeuchi S. (2008). Lipid bilayer microarray for parallel recording of transmembrane ion currents. Anal. Chem..

[41] Sandison M.E., Zagnoni M., Morgan H. (2007). Air-exposure technique for the formation of artificial lipid bilayers in microsystems. Langmuir.

[42] Malmstadt N., Nash M.A., Purnell R.F., Schmidt J.J. (2006). Automated formation of lipid-bilayer membranes in a microfluidic device. Nano Lett..

[43] Ota S., Suzuki H., Takeuchi S. (2011). Microfluidic lipid membrane formation on microchamber arrays. Lab Chip.

[44] Zagnoni M., Sandison M.E., Morgan H. (2009). Microfluidic array platform for simultaneous lipid bilayer membrane formation. Biosens. Bioelectron..

[45] Liu B.W., Rieck D., van Wie B.J., Cheng G.J., Moffett D.F., Kidwell D.A. (2009). Bilayer lipid membrane (BLM) based ion selective electrodes at the meso-, micro-, and nano-scales. Biosens. Bioelectron..

[46] Schibel A.E.P., Edwards T., Kawano R., Lan W.J., White H.S. (2010). Quartz nanopore membranes for suspended bilayer ion channel recordings. Anal. Chem..

[47] Fertig N., Klau M., George M., Blick R.H., Behrends J.C. (2002). Activity of single ion channel proteins detected with a planar microstructure. Appl. Phys. Lett..

[48] Suzuki H., Tabata K., Kato-Yamada Y., Noji H., Takeuchi S. (2004). Planar lipid bilayer reconstitution with a micro-fluidic system. Lab Chip.

[49] Orth A., Johannes L., Römer W., Steinem C. (2012). Creating and modulating microdomains in pore-spanning membranes. ChemPhysChem.

[50] Weiskopf D., Schmitt E.K., Klühr M.H., Dertinger S.K., Steinem C. (2007). Micro-BLMs on highly ordered porous silicon substrate: rupture process and lateral mobility. Langmuir.

[51] Nilsson J., Lee J.R.I., Ratto T.V., Letant S.E. (2006). Localized functionalization of single nanopores. Adv. Mater..

[52] Heyderman L.J., Ketterer B., Bächle D., Glaus F., Haas B., Schift H., Vogelsang K., Gobrecht J., Tiefenauer L., Dubochet O., Surbled P., Hessler T. (2003). High volume fabrication of customised nanopore membrane chips. Microelectron. Eng..

[53] Hirano-Iwata A., Aoto K., Oshima A., Taira T., Yamaguchi R.T., Kimura Y., Niwano M. (2010). Free-standing lipid bilayers in silicon chips—Membrane stabilization based on microfabricated apertures with a nanometer-scale smoothness. Langmuir.

[54] Peterman M.C., Ziebarth J.M., Braha O., Bayley H., Fishman H.A., Bloom D.M. (2002). Ion channels and lipid bilayer membranes under high potentials using microfabricated apertures. Biochem. Microdevices.

[55] Tiefenauer L., Studer A. (2008). Nano for bio: Nanopore arrays for stable and functionnal lipid bilayer membranes (Mini Review). Biointerphases.

[56] Kumar K., Isa L., Egner A., Schmidt R., Textor M., Reimhult E. (2011). Formation of nanopore-spanning lipid bilayers through liposome fusion. Langmuir.

[57] Hutter I., Müller E., Kristiansen P.M., Kresak S., Tiefenauer L. (2012). Polymer-based microfluidic device for measuring membrane protein activities. Microfluid. Nanofluid..

[58] Reimhult E., Kumar K. (2008). Membrane biosensor platforms using nano- and microporous supports. Trends Biotechnol..

[59] Worsfold O., Voelcker N.H., Nishiya T. (2006). Biosensing using lipid bilayers suspended on porous silicon. Langmuir.

[60] Hennesthal C., Drexler J., Steinem C. (2002). Membrane-suspended nanocompartments based on ordered pores in alumina. ChemPhysChem.

[61] Danelon C., Santschi C., Brugger J., Vogel H. (2006). Fabrication and functionalizuation of nanochannels by electron-beam-induced silicon oxide deposition. Langmuir.

[62] Studer A., Han X.J., Winkler F.K., Tiefenauer L.X. (2009). Formation of individual protein channels in lipid bilayers suspended in nanopores. Colloids Surfaces B.

[63] Ervin E.N., Kawano R., White R.J., White H.S. (2008). Simultaneous alternating and direct current readout of protein ion channel blocking events using glass nanopore membranes. Anal. Chem..

[64] Han X., Studer A., Sehr H., Geissbühler I., DiBerardino M., Winkler F.K., Tiefenauer L. (2007). Nanopore arrays for stable and functional free-standing lipid bilayers. Adv. Mater..

[65] Schmitt E.K., Vrouenraets M., Steinem C. (2006). Channel activity of OmpF monitored in nano-BLMs. Biophys. J..

[66] Römer W., Steinem C. (2004). Impedance analysis and single-channel recordings on nano-black lipid membranes based on porous alumina. Biophys. J..

[67] Gadsby D.C. (2004). Ion transport—Spot the difference. Nature.

[68] Studer A., Demarche S., Langenegger D., Tiefenauer L. (2011). Integration and recording of a reconstituted voltage-gated sodium channel in planar lipid bilayers. Biosens. Bioelectron..

[69] Fologea D., Gershow M., Ledden B., McNabb D.S., Golovchenko J.A., Li J.L. (2005). Detecting single stranded DNA with a solid state nanopore. Nano Lett..

[70] Davis R.W., Flores A. (2005). Nanoporous microbead supported bilayers: stability, physical characterization, and incorporation of functional transmembrane proteins. Langmuir.

[71] Sandison M.E., Zagnoni M., Abu-Hantash M., Morgan H. (2007). Micromachined glass apertures for artificial lipid bilayer formation in a microfluidic system. J. Micromech. Microeng..

[72] Kim P., Lee S.E., Jung H.S., Lee H.Y., Kawai T., Jeong H.E., Suh K.Y. Supported lipid bilayers microarrays onto a surface and inside microfluidic channels. Proceeding of 2006 International Conference on Microtechnologies in Medicine and Biology.

[73] Allbritton N.L. (2012). Micro total analyis systems for cell biology and biochemical assays. Anal. Chem..

[74] Gervais L., de Rooij N., Delamarche E. (2011). Microfluidic chips for point-of-care immunodiagnostics. Adv. Mater..

[75] Arora A., Simone G., Salieb-Beugelaar G.B., Kim J.T., Manz A. (2010). Latest developments in micro total analysis aystems. Anal. Chem..

[76] Fang Y., Frutos A.G., Lahiri J. (2002). Membrane protein microarrays. J. Am. Chem. Soc..

[77] Nakashima H., Furukawa K., Kashimura Y., Sumitomo K., Shinozaki Y., Torimitsu K. (2010). Pattern formation and molecular transport of histidine-tagged GFPs using supported lipid bilayers. Langmuir.

[78] Neumann J., Hennig M., Wixforth A., Manus S., Radler J.O., Schneider M.F. (2010). Transport, separation, and Accumulation of Proteins on Supported Lipid Bilayers. Nano Lett..

[79] Bally M., Bailey K., Sugihara K., Grieshaber D., Vörös J., Städler B. (2010). Liposome and lipid bilayer arrays towards biosensing applications. Small.

[80] Furukawa K., Aiba T. (2011). Supported lipid bilayer composition microarray fabricated by pattern-guided self-spreading. Langmuir.

[81] Vinchurkar M.S., Bricarello D.A., Lagerstedt J.O., Buban J.P., Stahlberg H., Oda N.M., Voss J.C., Parikh A.N. (2008). Bridging across length scales: multi-scale ordering of supported lipid bilayers via lipoprotein self-assembly and surface patterning. J. Am. Chem. Soc..

[82] Schift H. (2008). Nanoimprint lithography: An old story in modern times? A review. J. Vac. Sci. Technol. B.

[83] Zema L., Loreti G., Melocchi A., Maroni A., Gazzaniga A. (2012). Injection molding and its application to drug delivery. J. Control. Release.

[84] Gornall J.L., Mahendran K.R., Pambos O.J., Steinbock L.J., Otto O., Chimerel C., Winterhalter M., Keyser U.F. (2011). Simple reconstitution of protein pores in nano lipid bilayers. Nano Lett..

[85] Trietsch S.J., Hankemeier T., van der Linden H.J. (2011). Lab-on-a-chip technologies for massive parallel data generation in the life sciences: A review. Chemometr. Intell. Lab. Syst..

[86] Suzuki H., Le P.B., Takeuchi S. (2009). Ninety-six-well planar lipid bilayer chip for ion channel recording fabricated by hybrid stereolithography. Biomed. Microdevices.

[87] Ekins R.P. (1998). Ligand assays: From electrophoresis to miniaturized microarrays. Clin. Chem..

[88] Komolov K.E., Senin I.I., Philippov P.P., Koch K.W. (2006). Surface plasmon resonance study of G protein/receptor coupling in a lipid bilayer-free system. Anal. Chem..

[89] Müller D.J., Engel A. (2008). Strategies to prepare and characterize native membrane proteins and protein membranes by AFM. Curr. Opin. Colloid Interface Sci..

[90] Müller P., Rudin D.O., Tien H.T., Wescott W.C. (1962). Reconstitution of cell membrane structure *in vitro* and its transformation into an excitable system. Nature.

[91] Montal M., Müller P. (1972). Formation of biomolecular membranes from lipid monolayers and a study of their electrical properties. Proc . Natl Acad. Sci. USA.

[92] Läuger P. (1972). Carrier-mediated ion transport. Science.

[93] Kozuch J., Steinem C., Hildebrandt P., Millo D. (2012). Combined electrochemistry and surface-enhanced infrared absorption spectroscopy of gramicidin A incorporated into thethered bilayer lipid membranes. Angew. Chem. Int. Ed..

[94] Ovchinnikov Y.A. (1974). Membrane active complexones—Chemistry and biological function. FEBS Lett..

[95] Lingler S., Rubenstein I., Knoll W., Offenhäuser A. (1997). Fusion of small unilamellar lipid vesicles to alkanethiol and thiolipid self-assembled monolayers on gold. Langmuir.

[96] Puu G., Gustafson I. (1997). Planar lipid bilayers on solid supports from liposomes—Factors of importance for kinetics and stability. Biochim. Biophys. Acta.

[97] Hubbard J.B., Plant A.L. (1998). Self assembly driven by hydrophobic interactions at alkanethiol monolayers: Mechanism of formation of hybrid bilayer membranes. Biophys. Chem..

[98] Richter R.P., Brisson A. (2003). Characterization of lipid bilayers and protein assemblies supported on rough surfaces by atomic force microscopy. Langmuir.

[99] Reimhult E., Zäch M., Höök F., Kasemo B. (2006). A multitechnique study of liposome adsorption on Au and lipid bilayer formation on SiO_2_. Langmuir.

[100] Ratnayaka S.N., Wysockki R.J., Saavedra S.S. (2008). Preparation and characterization of asymmetric planar supported bilayers composed of poly(bis-sorbylphosphatidylcholine) on n-octadecyltrichlorosilane SAMs. J. Colloid Interface Sci..

[101] Zeineldin R., Last J.A., Slade A.L., Ista L.K., Bisong P., O'Brien M.J., Brueck S.R.J., Sasaki D.Y., Lopez G.P. (2006). Using bicellar mixtures to form supported and suspended lipid bilayers on silicon chips. Langmuir.

[102] Stamou D., Duschl C., Delamarche E., Vogel H. (2003). Self-assembled microarrays of attoliter molecular vessels. Angew. Chem. Int. Ed..

[103] Kim Y.H., Rahman M.M., Zhang Z.L., Misawa N., Tero R., Urisu T. (2006). Supported lipid bilayer formation by the giant vesicle fusion induced by vesicle-surface electrostatic attractive interaction. Chem. Phys. Lett..

[104] Rapuano R., Carmona-Ribeiro A.M. (2000). Supported bilayers on silica. J. Colloid Interface Sci..

[105] Bucak S., Wang C., Laibinis P.E., Hatton T.A. (2010). Dynamics of supported lipid bilayer deposition from vesicle suspensions. J. Colloid Interface Sci..

[106] Cross B., Steinberger A., Cottin-Bizonne C., Rieu J.P., Charlaix E. (2006). Boundary flow of water on supported phospholipid films. Europhys. Lett..

[107] Cho N.J., Frank C.W., Kasemo B., Höök F. (2010). Quartz crystal microbalance with dissipation monitoring of supported lipid bilayers on various substrates. Nat. Protoc..

[108] Simonsson L., Gunnarsson A., Wallin P., Jönsson P., Höök F. (2011). Continuous lipid bilayers derived from cell membranes for spatial molecular manipulation. J. Am. Chem. Soc..

[109] Jönsson P., Jonsson M.P., Tegenfeldt J.O., Höök F. (2008). A method improving the accuracy of fluorescence recovery after photobleaching analysis. Biophys. J..

[110] Mullineaux C.W., Kirchhoff H., Dopico A.M. (2007). Using fluorescence recovery after photobleaching to measure lipid diffusion in membranes. Methods in membrane lipids.

[111] Merzlyakov M., Li E., Hristova K. (2006). Directed assembly of surface-supported bilayers with transmembrane helixes. Langmuir.

[112] Berquand A., Mazeran P.E., Pantigny J., Proux-Delrouyre V., Laval J.M., Bourdillon C. (2003). Two-step formation of streptavidin-supported lipid bilayers by PEG-triggered vesicle fusion. Fluorescence and atomic force microscopy characterization. Langmuir.

[113] Seu K.J., Pandey A.P., Haque F., Proctor E.A., Ribbe A.E., Hovis J.S. (2007). Effect of surface treatment on diffusion and domain formation in supported lipid bilayers. Biophys. J..

[114] Almeida P.F.F., Vaz W.L.C., Lipowsky R., Sackmann E. (1995). Lateral diffusion in membranes. Handbook of Biological Physics.

[115] Benz M., Gutsmann T., Chen N.H., Tadmor R., Israelachvili J. (2004). Correlation of AFM and SFA measurements concerning the stability of supported lipid bilayers. Biophys. J..

[116] Seantier B., Breffa C., Felix O., Decher G. (2004). *In situ* investigations of the formation of mixed supported lipid bilayers close to phase transition temperature. Nano Lett..

[117] Jönsson P., Beech J.P., Tegenfeldt J.O., Höök F. (2009). Mechanical behavior of a supported lipid bilayer under external shear forces. Langmuir.

[118] Tien H.T., Ottova A.L. (1999). From self-assembled bilayer lipid membranes (BLMs) to supported BLMs on metal and gel substrates to practical applications. Colloids Surfaces A.

[119] Tanaka M., Sackmann E. (2006). Supported membranes as biofunctional interfaces and smart biosensor platforms. Phys. Status Solidi A.

[120] Tun T.N., Jenkins A.T.A. (2010). An electrochemical impedance study of the effect of pathogenic bacterial toxins on tethered bilayer lipid membrane. Electrochem. Commun..

[121] Sinner E.K., Knoll W. (2001). Functional tethered membranes. Curr. Oppi. Chem. Biol..

[122] Danelon C., Terrettaz S., Guenat O., Koudelka M., Vogel H. (2008). Probing the function of ionotropic and G protein-coupled receptors in surface-confined membranes. Methods.

[123] Terrettaz S., Mayer M., Vogel H. (2003). Highly electrically insulating tethered lipid bilayers for probing the function of ion channel proteins. Langmuir.

[124] Bunjes N., Schmidt E.K., Jonczyk A., Rippmann F., Beyer D., Ringsdorf H., Gräber P., Knoll W., Naumann R. (1997). Thiopeptide-supported lipid layers on solid substrates. Langmuir.

[125] Jadhav S.R., Sui D.X., Garavito R.M., Worden R.M. (2008). Fabrication of highly insulating tethered bilayer lipid membrane using yeast cell membrane fractions for measuring ion channel activity. J. Colloid Interface Sci..

[126] Stora T., Lakey J.H., Vogel H. (1999). Ion-channel gating in transmembrane receptor proteins: Functional activity in tethered lipid membranes. Angew. Chem. Int. Ed..

[127] Atanasov V., Knorr N., Duran R.S., Ingebrandt S., Offenhäuser A., Knoll W., Köper I. (2005). Membrane on a chip: A functional tethered lipid bilayer membrane on silicon oxide surfaces. Biophys. J..

[128] Raguse B., Braach-Maksvytis V., Cornell B.A., King L.G., Osman P.D.J., Pace R.J., Wieczorek L. (1998). Tethered lipid bilayer membranes: Formation and ionic reservoir characterization. Langmuir.

[129] Vockenroth I.K., Atanasova P.P., Long J.R., Jenkins A.T.A., Knoll W., Köper I. (2007). Functional incorporation of the pore forming segment of AChR M2 into tethered bilayer lipid membranes. Biochim. Biophys. Acta.

[130] Kügler R., Knoll W. (2002). Polyelectrolyte-supported lipid membranes. Bioelectrochemistry.

[131] Shen W.W., Boxer S.G., Knoll W., Curtis W.F. (2001). Polymer-supported bilayers on benzophenone-modified substrates. Biomacromolecules.

[132] Seitz M., Wong J.Y., Park C.K., Alcantar N.A., Israelachvili J. (1998). Formation of tethered supported bilayers via membrane-inserting reactive lipids. Thin solid films.

[133] Stenlund P., Babcock G.J., Sodroski J., Myszka D.G. (2003). Capture and reconstitution of G protein-coupled receptors on a biosensor surface. Anal. Biochem..

[134] Bockaert J. (2001). G protein-coupled receptors. Encyclopedia of Life Sciences.

[135] Claesson M., Frost R., Svedhem S., Andersson M. (2011). Pore spanning lipid bilayers on mesoporous silica having varying pore size. Langmuir.

[136] Kresak S., Hianik T., Naumann R.L.C. (2009). Giga-seal solvent-free bilayer lipid membranes: From single nanopores to nanopore arrays. Soft Matter.

[137] Tiefenauer L.X., Studer A. (2008). Nano for bio: Nanopore arrays for stable and functional lipid bilayer membranes (Mini Review). Biointerphases.

[138] Baba T., Toshima Y., Minamikawa H., Hato M., Suzuki K., Kamo N. (1999). Formation and characterization of planar lipid bilayer membranes from synthetic phytanyl-chained glycolipids. Biochim. Biophys. Acta.

[139] Heitz B.A., Jones I.W., Hall H.K., Aspinwall C.A., Saavedra S.S. (2010). Fractional polymerization of a suspended planar bilayer creates a fluid, highly stable membrane for ion channel recordings. J. Am. Chem. Soc..

[140] Jeon T.J., Malmstadt N., Poulos J.L., Schmidt J.J. (2008). Black lipid membranes stabilized through substrate conjugation to a hydrogel. Biointerphases.

[141] Sugihara K., Vörös J., Zambelli T. (2010). A Gigaseal obtained with a self-assembled long-lifetime lipid bilayer on a single polyelectrolyte multilayer-filled manopore. ACS Nano.

[142] Sugihara K., Vörös J., Zambelli T. (2010). The resistance of polyelectrolyte multilayers in a free-hanging configuration. J. Phys. Chem. B.

[143] Blake S., Capone R., Mayer M., Yang J. (2008). Chemically reactive derivatives of gramicidin A for developing ion channel-based nanoprobes. Bioconjugate Chem..

[144] Leonenko Z.V., Carini A., Cramb D.T. (2000). Supported planar bilayer formation by vesicle fusion: The interaction of phospholipid vesicles with surfaces and the effect of gramicidin on bilayer properties using atomic force microscopy. Biochim. Biophys. Acta.

[145] Bechinger B. (1997). Structure and function of channel-forming peptides: Maganinins, cecropin, melittin and alamethicin. J. Membr. Biol..

[146] Steinem C., Janshoff A., Galla H.J. (1998). Evidence for multilayer formation of melittin on solid-supported phospholipid membranes by shear-wave resonator measurements. Chem. Phys. Lipids.

[147] Strömstedt A.A., Wessman P., Ringstad L., Edwards K., Malmsten M. (2007). Effect of lipid headgroup composition on the interaction between melittin and lipid bilayers. J. Colloid Sci. Interfaces Sci..

[148] Favero G., Campanella L., D'Annibale A., Santucci R., Ferri T. (2003). Mixed hybrid bilayer lipid membrane incorporating valinomycin: Improvement in preparation and functioning. Microchem. J..

[149] Naumann R., Walz D., Schiller S.M., Knoll W. (2003). Kinetics of valinomycin-mediated K^+^ ion transport through tethered bilayer lipid membranes. J. Electroanal. Chem..

[150] Constantinescu I., Lafleur M. (2004). Influence of the lipid composition on the kinetics of concerted insertion and folding of melittin in bilayers. Biochim. Biophys. Acta.

[151] Shirai O., Yamana H., Ohnuki T., Yoshida Y., Kihara S. (2004). Ion transport across a bilayer lipid membrane facilitated by valinomycin. J. Electroanal. Chem..

[152] Iacovache I., Bischofberger M., van der Goot F.G. (2010). Structure and assembly of pore-forming proteins. Curr. Opin. Struct. Biol..

[153] Xiao H., Hong D.L., Zhu T.Y., Liu S.L., Li G.X. (2009). Electrochemical sensing of the ion-channel formation of OmpF. J. Appl. Electrochem..

[154] Hemmler R., Bose G., Wagner R., Peters R. (2005). Nanopore unitary permeability measured by electrochemical and optical single transporter recording. Biophys. J..

[155] Bayley H., Cremer P.S. (2001). Stochastic sensors inspired by biology. Nature.

[156] Baaken G., Ankri N., Schuler A.K., Rühe J., Behrends J.C. (2011). Nanopore-based single-molecule mass spectrometry on a lipid membrane microarray. ACS Nano.

[157] Bayley H., Jayasinghe L. (2004). Functional engineered channels and pores. Mol. Membr. Biol..

[158] Pennisi E. (2012). Search for pore-fection. Science.

[159] Bischofberger M., Gonzalez M.R., van der Goot F.G. (2009). Membrane injury by pore-forming proteins. Curr. Opin. Cell Biol..

[160] Schulte A., Ruamchan S., Khunkaewla P., Suginta W. (2009). The outer membrane protein VhOmp of *Vibrio harveyi:* Pore-forming properties in black lipid membranes. J. Membr. Biol..

[161] Manrao E.A., Derrington I.M., Laszlo A.H., Langford K.W., Hopper M.K., Gillgren N., Pavlenok M., Niederweis M., Gundlach J.H. (2012). Reading DNA at single-nucleotide resolution with a mutant MspA nanopore and phi29 DNA polymerase. Nat. Biotechnol..

[162] Holden M.A., Jayasinghe L., Daltrop O., Mason A., Bayley H. (2006). Direct transfer of membrane proteins from bacteria to planar bilayers for rapid screening by single-channel recording. Nat. Chem. Biol..

[163] Nurani G., Radford M., Charalambous K., O'Reilly A.O., Cronin N.B., Haque S., Wallace B.A. (2008). Tetrameric bacterial sodium channels: characterization of structure, stability, and drug binding. Biochemistry.

[164] Ozaki S., Aoki S., Hibi T., Kano K., Shirai O. (2008). Reconstitution of the voltage-gated K^+^ channel KAT1 in planar lipid bilayers. Electrochem. Commun..

[165] Karatekin E., Rothman J.E. (2012). Fusion of single proteoliposomes with planar, cushioned bilayers in microfluidic flow cells. Nat. Protoc..

[166] Kunze A., Svedhem S., Kasemo B. (2009). Lipid transfer between charged supported lipid bilayers and oppositely charged vesicles. Langmuir.

[167] Rawle R.J., van Lengerich B., Chung M., Bendix P.M., Boxer S.G. (2011). Vesicle fusion observed by content transfer across a tethered lipid bilayer. Biophys. J..

[168] de Planque M.R.R., Mendes G.P., Zagnoni M., Sandison M.E., Fisher K.H., Berry R.M., Watts A., Morgan H. (2006). Controlled delivery of membrane proteins to artificial lipid bilayers by nystatin-ergosterol modulated vesicle fusion. IED Proc. Nanobiotechnol..

[169] Woodbury D.J. (1999). Nystatin/ergosterol method for reconstituting ion channels into planar lipid bilayers. Methods Enzymol..

[170] Ataka K., Giess F., Knoll W., Naumann R., Haber-Pohlmeier S., Richter B., Heberle J. (2004). Oriented attachment and membrane reconstitution of His-tagged cytochrome c oxidase to a gold electrode: *In situ* monitoring by surface-enhanced infrared absorption spectroscopy. J. Am. Chem. Soc..

[171] Engelman D.M. (2005). Membranes are more mosaic than fluid. Nature.

[172] Trepot S., Mornet S., Benabdelhak H., Ducruix A., Brisson A., Lambert O. (2007). Membrane protein selectivity oriented on solid support and reconstituted into lipid membrane. Langmuir.

[173] White G.F., Racher K.I., Lipski A., Hallett F.R., Wood J.M. (2000). Physical properties of liposomes and proteoliposomes prepared from Escherichia coli polar lipids. Biochim. Biophys. Acta.

[174] Wenk R.M. (2005). The emerging field of lipidomics. Nat. Rev. Drug Discov..

[175] Lundbaek J.A., Birn P., Hansen A.J., Sogaard R., Nielsen C., Girshman J., Bruno M.J., Tape S.E., Egebjerg J., Greathouse D.V., Mattice G.L., Koeppe R.E., Andersen O.S. (2004). Regulation of sodium channel function by bilayer elasticity: The importance of hydrophobic coupling. Effects of micelle-forming amphiphiles and cholesterol. J. Gen. Physiol..

[176] Shenoy D.K., Barger W.R., Singh A., Panchal R.G., Misakian M., Stanford V.M., Kasianowicz J.J. (2005). Functional reconstitution of protein ion channels into planar polymerizable phospholipid membranes. Nano Lett..

[177] Ross E.E., Rozanski L.J., Spratt T., Liu S.C., O'Brien D.F., Saavedra S.S. (2003). Planar supported lipid bilayer polymers formed by vesicle fusion: 1. Influence of diene monomer structure and polymerization method on film properties. Langmuir.

[178] Subramaniam V., Alves I.D., Salgado G.F.J., Lau P.W., Wysocki R.J., Salamon Z., Tollin G., Hruby V.J., Brown M.F., Saavedra S.S. (2005). Rhodopsin reconstituted into a planar-supported lipid bilayer retains photoactivity after cross-linking polymerization of lipid monomers. J. Am. Chem. Soc..

[179] Eckford P.D.W., Sharom F.J. (2008). Interaction of the P-glycoprotein multidrug efflux pump with cholesterol: Effects on ATPase activity, drug binding and transport. Biochemistry.

[180] Simons K., Ikonen E. (1997). Functional rafts in cell membranes. Nature.

[181] Belli S., Elsener P.M., Wunderli-Allenspach H., Kramer S.D. (2009). Cholesterol-mediated activation of P-glycoprotein: Distinct effects on basal and drug-induced ATPase activities. J. Pharm. Sci..

[182] Lingwood D., Simons K. (2010). Model membrane to investigate lipid rafts as a membrane-organizing principle. Science.

[183] Crane J.M., Tamm L.K. (2004). Role of cholesterol in the formation and nature of lipid rafts in planar and spherical model membranes. Biophys. J..

[184] Fabre R.M., Okeyo G.O., Talham D.R. (2012). Supported lipid bilayers at skeletonized surfaces for the study of transmembrane proteins. Langmuir.

[185] Morigaki K., Kiyosue K., Taguchi T. (2004). Micropatterned composite membranes of polymerized and fluid lipid bilayers. Langmuir.

[186] Christensen S.M., Bolinger P.Y., Hatzakis N.S., Mortensen M.W., Stamou D. (2012). Mixing subattolitre volumes in a quantitative and highly parallel manner with soft matter nanofluidics. Nat. Nanotechnol..

[187] Lei G., MacDonald R.C. (2008). Effects on interactions of oppositely charged phospholipid vesicles of covalent attachment of polyethylene glycol oligomers to their surfaces: Adhesion, hemifusion, full fusion and “Endocytosis”. J. Membr. Biol..

[188] Luzio J.P., Pryor P.R., Bright N.A. (2007). Lysosomes: Fusion and function. Nat. Rev..

[189] Clejan S., Krulwich T.A., Mondrus K.R., Setoyoung D. (1986). Membrane lipid-composition of obligately and facultatively alkalophilic strains of bacillus SPP. J. Bacteriol..

[190] Baenziger J.E., Ryan S.E., Goodreid M.M., Vuong N.Q., Sturgeon R.M., DaCosta C.J.B. (2008). Lipid composition alters drug action at the nicotinic acetylcholine receptor. Mol. Pharmacol..

[191] Akesson A., Lind T., Ehrlich N., Stamou D., Wacklin H., Cardenas M. (2012). Composition and structure of mixed phospholipid supported bilayers formed by POPC and DPPC. Soft Matter.

[192] Braun P., LaBaer J. (2003). High throughput protein production for functional proteomics. Trends Biotechnol..

[193] Bleicher K.H., Böhm H.J., Müller K., Alanine A.I. (2003). Hit and lead generation: Beyond high-throughput screening. Nat. Rev. Drug Discov..

[194] Rask-Andersen M., Almen M.S., Schioth H.B. (2011). Trends in the exploitation of novel drug targets. Nat. Rev. Drug Discov..

[195] Warne T., Serrano-Vega M.J., Baker J.G., Moukhametzianov R., Edwards P.C., Henderson R., Leslie A.G.W., Tate C.G., Schertler G.F.X. (2008). Structure of a beta(1)-adrenergic G-protein-coupled receptor. Nature.

[196] Congreve M., Marshall F. (2010). The impact of GPCR structures on pharmacology and structure-based drug design. Bri. J. Pharmacol..

[197] Fang Y., Lahiri J., Picard L. (2003). G protein-coupled receptor microarrays for drug discovery. Drug Discov. Today.

[198] Swartz K.J. (2008). Sensing voltage across lipid membranes. Nature.

[199] Chakrapani S., Cuello L.G., Cortes D.M., Perozo E. (2008). Structural dynamics of an isolated voltage-sensor domain in a lipid bilayer. Structure.

[200] Zimmermann I., Dutzler R. (2011). Ligand activation of the prokaryotic pentameric ligand-gated ion channel ELIC. PLoS Biol..

[201] Debret G., Valadie H., Städler A.M., Etchebest C. (2008). New insights of membrane environment effects on MscL channel mechanics from theoretical approaches. Proteins Struct. Funct. Bioinforma..

[202] Sands Z.A., Sansom M.S.P. (2007). How does a voltage sensor interact with a lipid bilayer? Simulations of a potassium channel domain. Structure.

[203] Chanda B., Asamoah O.K., Blunck R., Roux B., Bezanilla F. (2005). Gating charge displacement in voltage-gated ion channels involves limited transmembrane movement. Nature.

[204] Decoursey T.E. (2003). Voltage-gated proton channels and other proton transfer pathways. Physiol. Rev..

[205] Gunthorpe N.J., Smith G.D., Davis J.B., Randall A.D. (2001). Characterisation of a human acid-sensing ion channel (hASIC1a) endogenously expressed in HEK293 cells. Pflügers Arch..

[206] Koishi R., Xu H., Ren D., Navarro B., Spiller B.W., Shi Q., Clapham D.E. (2004). A superfamily of voltage-gated sodium channels in bacteria. J. Biol.Chem..

[207] Shimizu H., Iwamoto M., Konno T., Nihei A., Sasaki Y.C., Oiki S. (2008). Global twisting motion of single molecular KcsA potassium channel upon Gating. Cell.

[208] Doyle D.A., Morais C.J., Pfuetzner R.A., Kuo A., Gulbis J.M., Cohen S.L., Chait B.T., MacKinnon R. (1998). The structure of the potassium channel: Molecular basis of K^+^ conduction and selectivity. Science.

[209] Shi N., Ye S., Alam A., Chen L., Jiang Y. (2006). Atomic structure of a Na^−^ and K^+^ conducting channel. Nature.

[210] Parekh A.B., Putney J.W. (2005). Store-operated calcium channels. Physiol. Rev..

[211] Singh H., Cousin M.A., Ashley R.H. (2007). Functional reconstitution of mammalian “chloride intracellular channels” CLIC1, CLIC4 and CLIC5 reveals differential regulation by cytoskeletal actin. FEBS J..

[212] Gwan J.F., Baumgaertner A. (2007). Cooperative transport in a potassium ion channel. J. Chem. Phys..

[213] Cordero-Morales J.F., Cuello L.G., Perozo E. (2006). Voltage-dependent gating at the KcsA selectivity filter. Nat. Struct. Mol. Biol..

[214] Shen R., Guo W.L. (2009). Ion binding properties and structure stability of the NaK channel. Biochim. Biophys. Acta.

[215] Nimigean C.M., Miller C. (2002). Na^+^ block and permeation in a K^+^ channel of known structure. J. Gen. Physiol.

[216] Yellen G. (2002). The voltage-gated potassium channels and their relatives. Nature.

[217] Dunlop J., Bowlby M., Peri R., Vasilyev D., Arias R. (2008). High-throughput electrophysiology: An emerging paradigm for ion-channel screening and physiology. Nat. Rev. Drug Discov..

[218] Leptihn S., Thompson J.R., Ellory J.C., Tucker S.J., Wallace M.I. (2011). *In vitro* reconstitution of eukaryotic ion channels using droplet interface bilayers. J. Am. Chem. Soc..

[219] Howitt S.M., Udvardi M.K. (2000). Structure, function and regulation of ammonium transporters in plants. Biochem. Biophys. Acta.

[220] Törnroth-Horsefield S., Wang Y., Hedfalk K., Johanson U., Karlsson M., Tajkhorshid E., Neutze R., Kjellbom P. (2006). Structural mechanism of plant aquaporin gating. Nature.

[221] Giacomini K.M., Huang S.M., Tweedie D.J., Benet L.Z., Brouwer K.L.R., Chu X.Y., Dahlin A., Evers R., Fischer V., Hillgren K.M., Hoffmaster K.A., Ishikawa T., Keppler D., Kim R.B., Lee C.A., Niemi M., Polli J.W., Sugiyama Y., Swaan P.W., Ware J.A., Wright S.H., Yee S.W., Zamek-Gliszczynski M.J., Zhang L., International T. (2010). Membrane transporters in drug development. Nat. Rev. Drug Discov..

[222] Demel M.A., Krämer O., Ettmayer P., Haaksma E.J., Ecker G.F. (2009). Predicting ligand interaction with ABC transporters in ADME. Chem. Biodivers..

[223] Jovanovic-Talisman T., Tetenbaum-Novatt J., McKenney A.S., Zilman A., Peters R., Rout M.P., Chait B.T. (2009). Artificial nanopores that mimic the transport selectivity of the nuclear pore complex. Nature.

[224] Lim R.Y.H., Aebi U., Fahrenkrog B. (2008). Towards reconciling structure and function in the nuclear pore complex. Histochem. Cell. Biol..

[225] Ren D., Navarro B., Xu H., Yue L., Shi Q., Clapham D.E. (2001). A prokaryotic voltage-gated sodium channel. Science.

[226] Ito M., Xu H., Guffanti A.A., Wei Y., Zvi L., Clapham D.E., Krulwich T.A. (2004). The voltage-gated Na^+^ channel NavBP has a role in motility, chemotaxis and pH homeostasis of an alkaliphilic Bacillus. Proc. Nat. Acad. Sci. USA.

[227] Shafrir Y., Durell S.R., Guy H.R. (2008). Models of the structure and gating mechanisms of the pore domain of the NaChBac ion channel. Biophys. J..

[228] Ogiwara I., Miyamoto H., Morita N., Atapour N., Mazaki E., Inoue I., Takeuchi T., Itohara S., Yanagawa Y., Obata K., Furuichi T., Hensch T.K., Yamakawa K. (2007). Na(v)1.1 localizes to axons of parvalbumin-positive inhibitory interneurons: A circuit basis for epileptic seizures in mice carrying an Scn1a gene mutation. J. Neurosci..

[229] Auerbach A. (2003). Life at the top: The transition state of AChR gating. Sci. STKE.

[230] Corringer P.J., Poitevin F., Prevost M.S., Sauguet L., Delarue M., Changeux J.P. (2012). Structure and pharmacology of pentameric receptor channels: From bacteria to brain. Structure.

[231] Liu X.L., Xu Y.C., Li H.L., Wang X.C., Jiang H.L., Barrantes F.J. (2008). Mechanics of channel gating of the nicotinic acetylcholine receptor. PLoS Comput. Biol..

[232] Hilf R.J.C., Dutzler R. (2009). Structure of a potentially open state of a proton-activated pentameric ligand-gated ion channel. Nature.

[233] Engel A., Gaub H.E. (2008). Structure and mechanism of membrane proteins. Ann. Rev. Biochem..

[234] Friedrich M.G., Robertson J.W.F., Walz D., Knoll W., Naumann R.L.C. (2008). Electronic wiring of a multi-redox site membrane protein in a biomimetic surface architecture. Biophys. J..

[235] Sperotto M.M., May S., Baumgaertner A. (2006). Modelling of proteins in membranes. Chem. Phys. Lipids.

[236] DeWire S.M., Ahn S., Lefkowitz R.J., Shenoy S.K. (2007). Beta-arrestins and cell signaling. Ann. Rev. Physiol..

[237] Picas L., Rico F., Scheuring S. (2012). Direct measurement of the mechanical properties of lipid phases in supported bilayers. Biophys. J..

[238] Ovalle-Garcia E., Torres-Heredia J.J., Antillon A., Ortega-Blake I. (2011). Simultaneous determination of the elastic properties of the lipid bilayer by atomic force microscopy: Bending, tension, and adhesion. J. Physical Chem. B.

[239] Steltenkamp S., Müller M.M., Desemo M., Hennesthal C., Steinem C., Janshoff A. (2006). Mechanical properties of pore-spanning lipid bilayers probed by atomic force microscopy. Biophys. J..

[240] Li J.K., Sullan R.M.A., Zou S. (2011). Atomic force microscopy force mapping in the study of supported lipid bilayers. Langmuir.

[241] Charalambous K., Booth P.J., Woscholski R., Seddon J.M., Templer R.H., Law R.V., Barter L.M.C., Ces O. (2012). Engineering de novo membrane-mediated protein-protein communication networks. J. Am. Chem. Soc..

[242] Veglia G., Ramamoorthy A. (2010). Special issue on “membrane protein dynamics: Correlating structure to function”. Biochim. Biophys. Acta.

[243] Drews J. (2000). Drug discovery: A historical perspective. Science.

[244] Wood C., Williams C., Waldron G.J. (2004). Patch clamping by numbers. Drug Discov. Today.

[245] Masetti M., Cavalli A., Recanatini M. (2008). Modeling the hERG potassium channel in a phospholipid bilayer: Molecular dynamics and drug docking studies. J. Comput. Chem..

[246] Zou B.Y., Yu H.B., Babcock J.J., Chanda P., Bader J.S., McManus O.B., Li M. (2010). Profiling diverse compounds by flux- and electrophysiology-based primary screens for inhibition of human ether-a-go-go related gene potassium channels. Assay Drug Dev. Technol..

[247] Schmalhofer W.A., Swensen A.M., Thomas B.S., Felix J.P., Haedo R.J., Solly K., Kiss L., Kaczorowski G.J., Garcia M.L. (2010). A pharmacologically validated, high-capacity, functional thallium flux assay for the human ether-à-go-go related gene potassium channel. Assay Drug Dev. Technol..

[248] Poulos J.L., Jeon T.J., Damoiseaux R., Gillespie E.J., Bradley K.A., Schmidt J.J. (2009). Ion channel and toxin measurement using a high throughput lipid membrane platform. Biosens. Bioelectron..

[249] Siontorou C.G., Batzias F.A. (2010). Innovation in biotechnology: Moving from academic research to product development-the case of biosensors. Crit. Rev. Biotechnol..

[250] Lodowski D.T., Angel T.E., Palczewski K. (2009). Comparative analysis of GPCR crystal structures. Photochem. Photobiol..

[251] Jones P.M., George A.M. (2004). The ABC transporter structure and mechanism: Perspectives on recent research. Cell. Mol. Life Sci..

[252] Landau E.M., Rosenbusch J.P. (1996). Lipidic cubic phases: A novel concept for the crystallization of membrane proteins. Proc. Nat. Acad. Sci. USA.

[253] Haupts U., Tittor J., Oesterhelt D. (1999). Closing in on bacteriorhodopsin: Progress in understanding the molecule. Ann. Rev. Biophysics Biomol. Struct..

[254] Palczewski K., Kumasaka T., Hori T., Behnke C.A., Motoshima H., Fox B.A., Le Trong I., Teller D.C., Okada T., Stenkamp R.E., Yamamoto M., Miyano M. (2000). Crystal structure of rhodopsin: A G protein-coupled receptor. Science.

[255] Standfuss J., Edwards P.C., D'Antona A., Fransen M., Xie G.F., Oprian D.D., Schertler G.F.X. (2011). The structural basis of agonist-induced activation in constitutively active rhodopsin. Nature.

[256] Tate C.G., Schertler G.F.X. (2009). Engineering G protein-coupled receptors to facilitate their structure determination. Curr. Opin. Struct. Biol..

[257] Dahmane T., Damian M., Mary S., Popot J.L., Baneres J.L. (2009). Amphipol-assisted *in vitro* folding of G protein-coupled receptors. Biochemistry.

[258] Bayburt T.H., Sligar S.G. (2010). Membrane protein assembly into nanodiscs. FEBS Lett..

[259] Gawrisch K., Soubias O., Mihailescu M. (2008). Insights from biophysical studies on the role of polyunsaturated fatty acids for function of G-protein coupled membrane receptors. Prostagland. Leuk. Essent. Fat. Acids.

[260] Jonsson M.P., Jönsson P., Dahlin A.B., Höök F. (2007). Supported lipid bilayer formation and lipid-membrane-mediated biorecognition reactions studied with a new nanoplasmonic sensor template. Nano Lett..

[261] Zhao J., Tamm L. (2003). FTIR and fluorescence studies of interaction of synaptic fusion proteins in polymer-supported bilayers. Langmuir.

[262] Kundu J., Levin C.S., Halas N.J. (2009). Real-time monitoring of lipid transfer between vesicles and hybrid bilayers on Au nanoshells using surface enhanced Raman scattering (SERS). Nanoscale.

[263] Frederix P.L.T.M., Bosshart P.D., Engel A. (2009). Atomic force microscopy of biological membranes. Biophys. J..

[264] Tanaka M., Sackmann E. (2005). Polymer-supported membranes as models for the cell surface. Nature.

